# Pointed-end processive elongation of actin filaments by *Vibrio* effectors VopF and VopL

**DOI:** 10.1126/sciadv.adc9239

**Published:** 2022-11-18

**Authors:** Elena Kudryashova, Ankita, Heidi Ulrichs, Shashank Shekhar, Dmitri S. Kudryashov

**Affiliations:** ^1^ Department of Chemistry and Biochemistry, The Ohio State University, Columbus, OH 43210, USA.; ^2^ Department of Physics, Emory University, Atlanta, GA 30322, USA.; ^3^ Department of Cell Biology, Emory University, Atlanta, GA 30322, USA.

## Abstract

According to the cellular actin dynamics paradigm, filaments grow at their barbed ends and depolymerize predominantly from their pointed ends to form polar structures and do productive work. We show that actin can elongate at the pointed end when assisted by *Vibrio* VopF/L toxins, which act as processive polymerases. In cells, processively moving VopF/L speckles are inhibited by factors blocking the pointed but not barbed ends. Multispectral single-molecule imaging confirmed that VopF molecules associate with the pointed end, actively promoting its elongation even in the presence of profilin. Consequently, VopF/L can break the actin cytoskeleton’s polarity by compromising actin-based cellular processes. Therefore, actin filament design allows processive growth at both ends, which suggests unforeseen possibilities for cellular actin organization, particularly in specialized cells and compartments.

## Teaser

*Vibrio* VopF/L toxins challenge the classic actin treadmilling paradigm by promoting unconventional minus-end actin elongation.

## INTRODUCTION

Polarity is an inherent characteristic of actin filaments (F-actin) manifested by a faster net association of actin monomers (G-actin) at the plus (barbed) end and their faster net dissociation at the minus (pointed) end (*1*). Pointed-end elongation is disfavored by the structural properties of the actin filament pointed end (*2*). In cells, this disparity between filament ends is exacerbated by actin-binding proteins (ABPs). As an example, G-actin binding protein profilin (PFN) prohibits monomer addition at the pointed end (*3*) but enables their association at the barbed end while also assisting in accelerated elongation by formins (*4*) and other proteins. ADF/cofilins, on the other hand, can sense aged, adenosine diphosphate (ADP)–enriched filaments and, together with partner proteins, promote filament severing and pointed-end depolymerization (*5*–*7*).

Directional elongation of polar filaments enables higher-order polarity of branched and bundled actin assemblies, generating force required for membrane deformation upon cell migration, endocytosis, autophagy, mitochondrial fission, and other events (*8*–*10*). Because cellular conditions disfavor spontaneous nucleation, the directionality of all newly formed filaments is governed by carefully controlled localization of the nucleation and plus-end assembly factors and/or their activators. Examples of such factors localized at the cytoplasmic or organelle membranes include nucleation-promoting factors activating the branched actin polymerization by the Arp2/3 complex (*11*), linear filament nucleators/processive motors formins (*12*, *13*), and tandem Wiskott-Aldrich syndrome protein homology 2 (WH2) nucleators Spire (*14*) and Cobl (*15*). While the long-range transport in eukaryotic cells almost entirely relies on microtubule-dependent motors, directional orientation of actin filaments at the cell cortex and other peripheral compartments (e.g., in the lamellipodia, filopodia, stereocilia, microvilli, and pseudopods) enables short-range directional cargo transport mediated by plus-end– or minus-end–directed myosin motors [e.g., myosins V, VI, IX, and X (*16*)] and contributes to membrane tension generation by single-headed myosins [e.g., myosin I (*17*)]. Because the cellular actin machinery is designed to establish and promote polarity, no host factors are currently recognized to promote polymerization at the pointed ends. The resulting polarity of the actin cytoskeleton contributes to numerous physiological processes, including but not limited to the organelle biogenesis (*18*–*20*), establishing the planar (*21*) and basal-apical (*22*) cell polarity and right-left asymmetry in embryogenesis (*23*), conferring the integrity of epithelial barriers (*24*) and the innate immune response (*25*).

**Fig. 1.**
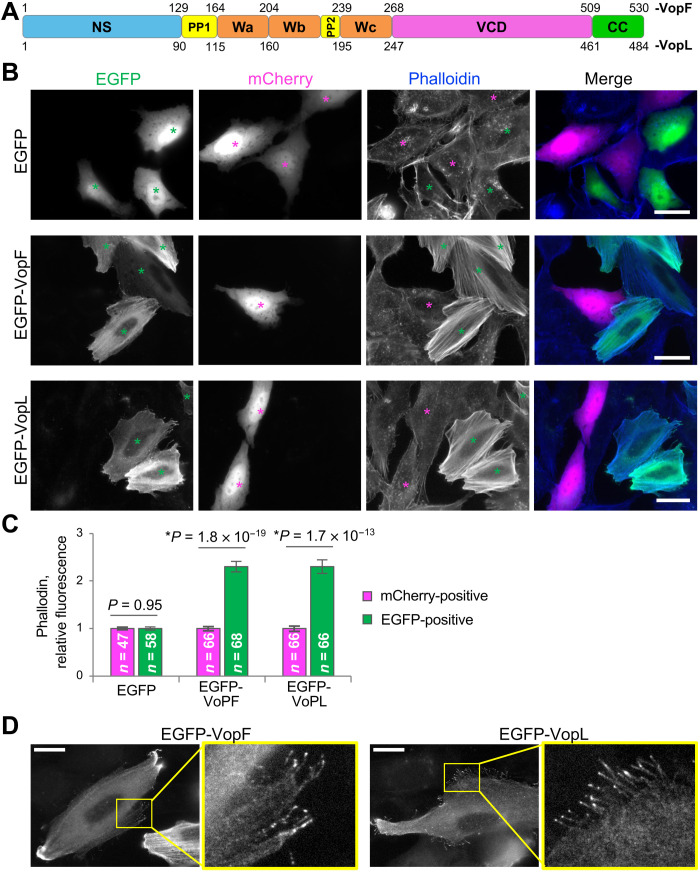
VopF and VopL induce excessive accumulation of F-actin and formation of thin protrusions in transfected cells. (**A**) Domain structure of VopF/L: NS, N-terminal sequence; PP1 and PP2, polyproline-rich regions; Wa, Wb, and Wc, WH2 domains; VCD, Vop C-terminal domain; CC, C-terminal dimerization coiled coil. Amino acid numbering is shown for both VopF and VopL. (**B** and **C**) Analysis of F-actin content by phalloidin staining in U2OS cells expressing moderate levels of enhanced green fluorescent protein (EGFP)–tagged VopF or VopL. (B) Representative images of mixed populations of U2OS cells transiently transfected with EGFP, EGFP-VopF, or EGFP-VopL co-plated with mCherry-transfected U2OS cells serving as an internal control in each image. Cocultures were fixed and stained for F-actin by coumarin-phalloidin. Green and magenta asterisks denote EGFP- and mCherry-positive cells, respectively. Scale bars, 30 μm. (C) Coumarin-phalloidin fluorescence was quantified for EGFP- and mCherry-positive cells in each image and normalized to the control level in the mCherry-positive cells. Numerical data presented as means ± SEM; *n* is the number of cells. Student’s *t* test was applied to compare two groups (EGFP- and mCherry-positive) for each EGFP construct. (**D**) Representative images of cellular protrusions in U2OS cells expressing moderate levels of EGFP-tagged VopF or VopL. Yellow boxes are zoomed. Scale bars, 20 μm.

Hence, hijacking or disrupting the polarity of the actin cytoskeleton is beneficial for bacterial pathogens and can be achieved by different means (*26*, *27*). *V. cholerae* and *V. parahaemolyticus* are pathogens from the *Vibrio* genus that achieve this goal by producing type III secretion system VopF and VopL toxins. Both toxins are important pathogen survival factors that work by disrupting actin homeostasis (*28*–*31*). VopF is required for efficient colonization of *V. cholerae* in the infant mouse model (*30*, *31*), while VopL contributes to the intracellular survival of *V. parahaemolyticus* by establishing and maintaining a “protective intracellular replicative niche” (*28*) via mysterious mechanisms. VopF/L are homologous proteins (71% similarity) that share their domain organization (Fig. 1A). N-terminal sequence (NS) is responsible for the toxin translocation into a host cell but does not participate in actin assembly (*32*, *33*). The presence of three tandem WH2 domains (e.g., similar to those found in host actin WH2 nucleators Spire and Cobl) (*34*) denoted the toxins’ capacity to nucleate actin. When compared side by side in reconstituted total internal reflection fluorescence microscopy (TIRFM) experiments (*32*), both toxins were shown to be pointed-end nucleators, while VopF/L brief association with the preexisting barbed ends and an inferred involvement in their elongation (*35*) was later suggested to be nonphysiological (*32*). Dimerization of the Vop C-terminal domain (VCD) of VopL through the C-terminal coiled-coil (CC) stabilizes lateral (short-pitch) contacts between actin monomers to create a pointed-end nucleus (*33*, *36*, *37*), while the high-affinity WH2 domains contribute to the nucleation by bringing monomers for the VCD and dissociate upon nucleation (*32*, *37*). The pointed-end association of the toxins was reported to be rather weak, leading to fast cycles of filament nucleation and detachments with minimal to no effect on actin elongation in vitro: VopF/L-nucleated filaments elongated at the barbed ends at the control barbed-end growth rate (*32*, *33*). No pointed-end elongation was observed, and it was assumed that monomer incorporation at the pointed ends is blocked when VopF/L are bound.

In the present study, observations of VopF and VopL intracellular behavior at the single-molecule level within physiological context prompted us to revisit their properties using in vitro reconstituted conventional and microfluidics-assisted (mf)–TIRFM assays. Combined, our results demonstrate that (i) under physiological conditions actin filaments can be elongated processively at the pointed end and (ii) VopF/L toxins are founding members of the actin pointed-end polymerase family.

## RESULTS

### VopF/L induce similar cellular phenotypes: Excessive accumulation of F-actin and induction of thin protrusions

Despite their similar abilities to nucleate actin at the pointed end, VopF and VopL were reported to cause different cellular phenotypes. They were, however, yet to be compared side by side in a single study, keeping possibilities for alternative interpretations. To address this uncertainty, we transiently transfected human U2OS osteoblast cells with enhanced green fluorescent protein (EGFP), EGFP-VopF, or EGFP-VopL and co-plated them with mCherry-transfected U2OS cells used as an internal negative control to compare the content of F-actin stained with fluorescently labeled phalloidin (Fig. 1B). In agreement with their reported actin nucleation activity, both bacterial effectors induced prolific stress fibers, increasing F-actin content 2.3 times compared to control cells (Fig. 1, B and C), the effect reported previously for VopL only (*29*). The F-actin promoting effect was not cell type specific, as it was also observed in *Xenopus laevis* fibroblast (XTC) cells (fig. S1). Thus, despite a lack of pronounced stress fibers in the VopF/L-transfected spreading (i.e., imaged at 30 min after plating) XTC cells, the phalloidin staining was notably denser compared to nontransfected cells (fig. S1, A to D). Furthermore, 24 hours after plating, abundant VopF/L-induced stress fibers accumulated in XTC cells (fig. S1E), similar to those in the transfected U2OS cells but not in control cells of either type.

Notably, in addition to the accumulation of F-actin, both toxins induced thin, flexible actin-positive protrusions emanating from the cell edge, often nonattached to the substrate, moderately enriched with VopF/L at the tips (Fig. 1D; fig. S1, C and D; and movies S1 and S2). This activity has been previously recognized exclusively for VopF (*30*, *31*). Morphologically, the observed VopF/L-induced protrusions were reminiscent of those formed by a constitutively active diaphanous mDia2 formin (movie S2) capable of barbed-end actin elongation (*38*, *39*). This similarity prompted us to explore whether VopF/L have an actin elongation activity.

### VopF/L move processively inside the cell in an actin-dependent manner

**Fig. 2.**
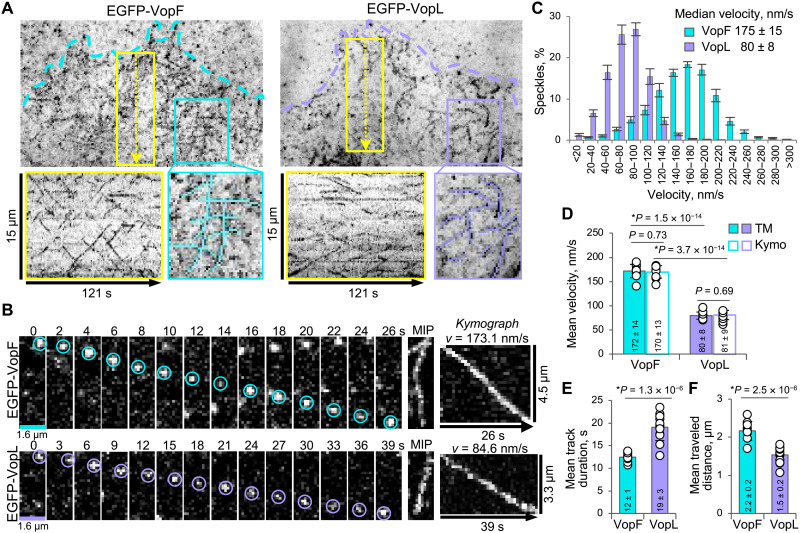
VopF and VopL move processively inside the cell. (**A**) Transiently transfected spreading XTC cells expressing very low (i.e., suitable for single-molecule analysis) levels of EGFP-tagged VopF or VopL were selected for TIRF SiMS imaging. Maximum intensity projections of time-lapse images show trajectories of moving EGFP-VopF and EGFP-VopL speckles. Dashed lines outline the cell edge. Boxed areas (cyan for VopF and violet for VopL) are zoomed below to show the projected tracks (outlined in the zoomed views). Kymographs obtained from the 30-pixel-wide lanes (yellow boxes with dotted arrows) are shown in yellow boxes below; yellow dotted arrows also serve as scale bars (15 μm). (**B**) TIRF SiMS time-lapse images show processively moving SiMSs of EGFP-VopF and EGFP-VopL. Maximum intensity projections (MIP) and kymographs obtained from drawing segmented lines through the speckle trajectories are shown. (**C** to **F**) TrackMate analysis of processively moving VopF/L single particles: (C) Velocity distribution (histogram), (D) comparison of mean velocities calculated using TrackMate and kymograph analyses, (E) average track duration, and (F) average track length. Numerical data presented as means ± SEM [for (C)] and means ± SD [for (D) to (F)]; *n* is 10 and 12 cells for VopF and VopL, respectively.

To better understand the VopF/L intracellular behavior, we examined them using high-resolution TIRF single-molecule speckle (SiMS) live-cell microscopy (*40*). One of the best and most characterized model systems for SiMS microscopy is XTC cells expressing low levels of EGFP-tagged protein of interest under defective cytomegalovirus (dCMV) promoter (*41*). EGFP-tagged VopF and VopL molecules were visualized as fluorescent speckles (movie S3), suggesting their association with large cellular structures (e.g., the cytoskeleton) limiting their diffusion. Automatic tracking of VopF/L single-molecule particles using Fiji (*42*) plug-in TrackMate (*43*, *44*) revealed stationary and motile speckle populations (fig. S2, A to F, and movie S4). More than 40% of the VopF/L speckles were reliably identified by automatic tracking as moving on linear trajectories for at least three consecutive frames (i.e., 6 s), suggesting their processive propulsion by a motor (Fig. 2, fig. S2, and movie S4). VopF moved ~2 times faster than VopL (172 versus 80 nm/s). In comparison, these rates are at least an order of magnitude slower than that of mDia1 formin [1.8 to 2 μm/s (*38*, *45*)] but several-fold faster than the lamellipodial actin retrograde flow rates [25 nm/s (*40*)].

In eukaryotic cells, protein machines capable of producing directed linear translocations include F-actin, microtubules, and their associated motor proteins (myosins, kinesins, and dynein). The observed intracellular movement of VopF/L did not depend on myosin or microtubules/microtubule-associated motors, as it was not inhibited by myosin inhibitors (blebbistatin and MyoVin1) or microtubule-depolymerizing drug nocodazole (fig. S3, A and B, and movie S5). In contrast, the movement was effectively inhibited by actin-targeting drugs (namely, jasplakinolide, cytochalasin D, swinholide A, and latrunculin A) at micromolar concentrations (movie S6 and fig. S3C), suggesting that the cellular motility of the toxins is coupled to actin polymerization at a filament end. The VopF/L speckles assigned as stationary or moving randomly (~60% of total population automatically tracked by TrackMate software) may represent (i) molecules associated with other cellular structures (e.g., via the NS) (*31*) and (ii) misfolded or otherwise nonfunctional molecules due to ectopic expression, partial proteolytic digestion, or photodamage.

Previous in vitro studies showed that VopF/L function as homodimers (*32*, *33*, *36*, *37*). To determine the oligomerization state of VopF/L in the intracellular speckles, we monitored changes in their fluorescence intensities upon photobleaching (*46*). The analysis of the stepwise photobleaching profiles suggested the dimeric state for both VopF and VopL intracellular particles (fig. S4). Therefore, to stay associated with filament growing ends, VopF/L do not require a high-order oligomerization [e.g., as vasodilator-stimulated phosphoprotein (VASP)] (*47*) but function as homodimers (e.g., as formins).

### WH2 domains enable processive motility of the dimeric VCD

**Fig. 3.**
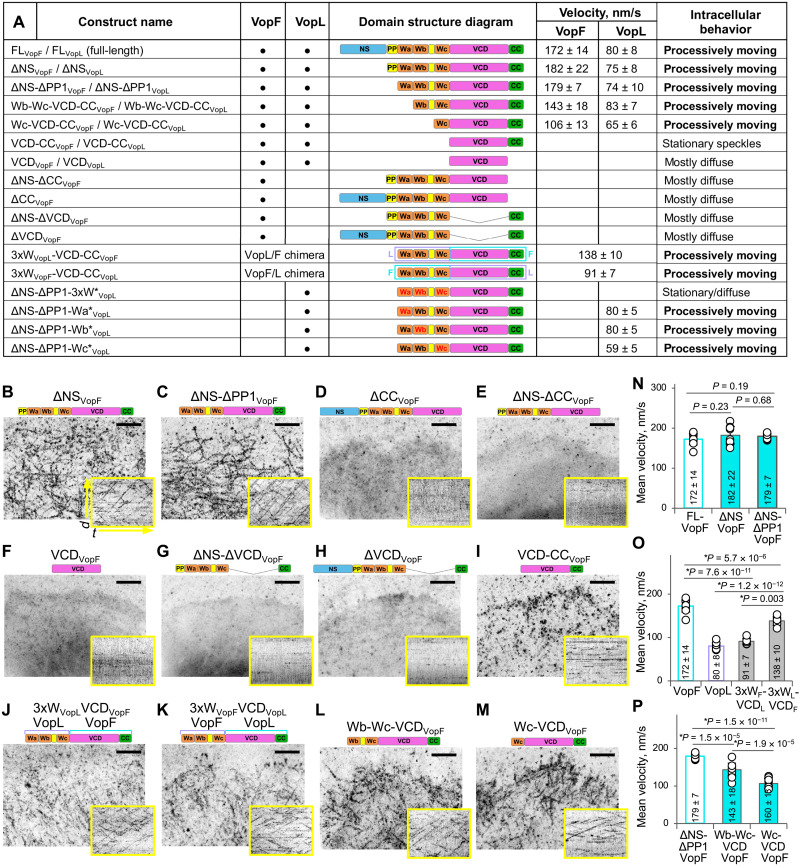
At least one WH2 domain is necessary in addition to the dimeric VCD to support the intracellular processive motility of VopF/L. (**A**) VopF/L constructs used in this study. WH2 domains in red font contain mutations abolishing actin binding. Velocities of the processively moving constructs [combined numerical data from (N) to (P) and fig. S5 (K to M) presented as means ± SD]. (**B** to **M**) Transiently transfected spreading XTC cells expressing very low (i.e., suitable for single-molecule analysis) levels of EGFP-tagged constructs were selected for TIRF SiMS imaging. Maximum intensity projections of time-lapse images show trajectories of moving speckles (B, C, and J to M), stationary speckles (I), or mainly diffused localization (D to H). Scale bars, 5 μm. Kymographs are shown in yellow boxes; *d* is distance (15 μm), and *t* is time (121 s). (**N** to **P**) Mean velocities of processively moving constructs; numerical data presented as means ± SD. Number of cells: ΔNS-VopF, *n* = 11; ΔNS-ΔPP1-VopF, *n* = 9; Wb-Wc-VCD-VopF, *n* = 12; Wc-VCD-VopF, *n* = 11; 3xW-VopL/VCD-VopF chimera, *n* = 10; 3xW-VopF/VCD-VopL chimera, *n* = 8.

Analysis of a series of deletion VopF/L mutants (Fig. 3A) revealed that the NS of VopF/L along with the first N-terminally located polyproline (PP)–rich sequence (PP1) is dispensable for the processive movement of the constructs (Fig. 3, B, C, and N, and fig. S5, A, B, and K). In contrast, deletion of the C-terminal dimerization CC resulted in mostly diffused localization of the ΔCC constructs (Fig. 3, D to F, and fig. S5F), suggesting that the CC-dependent dimerization is required for the intracellular processive movement of VopF/L just as for actin nucleation observed in vitro (*33*, *36*, *37*).

However, in the absence of WH2 domains, dimerization of the VCD was sufficient for stable association with the cytoskeleton but insufficient to support the processive movement (Fig. 3I and fig. S5E), while dimerization of WH2 domains in the absence of VCD supported neither the movement nor stable association with the cytoskeleton (Fig. 3, G and H).

To address the source of the velocity rate difference between VopF/L, we created chimeric constructs by swapping the VCD and tandem WH2 domains in the Vop orthologs (3xW_VopL_VCD_VopF_ and 3xW_VopF_VCD_VopL_; Fig. 3, A, J, and K). The chimeras displayed intermediate velocities (compared to those of the original Vop constructs; Fig. 3O), which were, however, notably closer to those of the parental wild-type (WT) Vop proteins, from which the VCD domains were originated (138 nm/s 3xW_VopL_VCD_VopF_ versus 172 nm/s WT VopF and 91 nm/s 3xW_VopF_VCD_VopL_ versus 80 nm/s WT VopL). Therefore, while both VCD and WH2 domains contribute to the VopF/L velocity, VCD is its major determinant.

Deletion of either the first (Wa) or the first two (Wa and Wb) WH2 domains of VopF preserved the processive movement but resulted in the reduced speckle velocities overall proportional to the number of the removed WH2 domains (Fig. 3, A, L, M, and P, and fig. S5, C, D, and L). In VopL, compromising the actin-binding properties of the first two WH2 domains (Wa and Wb) by mutating blocks of four highly conserved residues (*29*) similarly preserved the ability of the constructs to move processively but had no measurable effect on the velocity (fig. S5, H, I, and M). However, the analogous mutations in the WH2 domain closest to VCD (Wc) decreased the velocity (fig. S5, J and M). Mutating all three WH2 domains of VopL resulted in the immotile speckles (fig. S5G), corroborating the results with the removal of all three WH2 domains from both VopF and VopL (VCD-CC; Fig. 3I and fig. S5E). Therefore, at least one functional WH2 domain is required to support the intracellular processive movement of VopF/L.

### VopF/L directional movement is affected by blocking F-actin pointed but not barbed ends

**Fig. 4.**
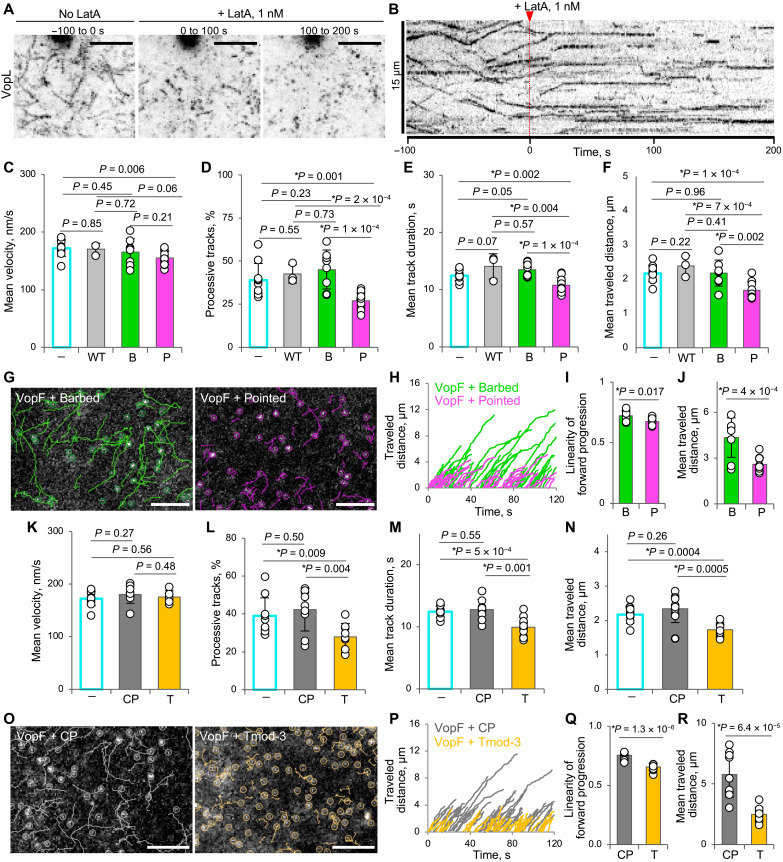
Blocking F-actin pointed ends interferes with VopF/L directional movement. (**A** and **B**) Addition of 1 nM latrunculin A (LatA) abolishes processive motility of VopL. Transiently transfected spreading XTC cells expressing very low (i.e., suitable for single-molecule analysis) levels of EGFP-VopL were selected for TIRF SiMS imaging. Maximum intensity projections (A) and kymograph (B) of TIRF SiMS time-lapse images taken before (−100 to 0 s) and immediately after the addition of LatA (0 to 100 s and 100 to 200 s). Scale bars, 5 μm (A). LatA was added at time “0” (marked on the kymograph by red arrow). (**C** to **J**) Coexpression of EGFP-VopF with either wild-type actin (“WT”) or actin mutants with disrupted barbed (“B”) or pointed (“P”) surfaces. Cells expressing both moderate to low levels of mCherry-tagged actin constructs and very low (i.e., suitable for single-molecule analysis) levels of EGFP-VopF were selected for TIRF SiMS analysis. Numerical data are presented as means ± SD. Number of cells: barbed-surface mutant, *n* = 8; pointed-surface mutant, *n* = 12. (G) Representative images showing 50 longest VopF tracks (scale bar, 5 μm); (H) the same tracks plotted as traveled distance as a function of time; (I and J) linearity of forward progression (i.e., the ratio between the mean straight line speed and the track mean speed) and average track length calculated for the longest 50 tracks in all cells. (**K** to **R**) Coexpression of EGFP-VopF with either capping protein (“CP”) or Tmod-3 (“T”). Cells expressing both moderate to low levels of mCherry-tagged CP or Tmod-3 and very low (i.e., suitable for single-molecule analysis) levels of EGFP-VopF were selected for TIRF SiMS analysis. Numerical data presented as means ± SD. Number of analyzed cells: CP, *n* = 9; Tmod-3, *n* = 10. (O to R) Analysis of 50 longest VopF tracks upon coexpression with either CP or Tmod-3 [same as (G) to (J)].

To test whether VopF/L processive motility is associated with the barbed or pointed filament end, we first used drugs affecting actin via different mechanisms: Jasplakinolide stabilizes F-actin (*48*, *49*), swinholide A binds to actin dimers and severs actin filaments (*50*), and cytochalasin D (*51*, *52*) and latrunculin A (*53*, *54*) sequester actin monomers by binding to their “barbed” or “pointed” surfaces, respectively. While at micromolar concentrations all the drugs showed inhibitory effects (movie S6), at nanomolar concentrations only latrunculin A potently arrested the VopF/L motility within seconds upon the drug addition (Fig. 4, A and B, and movies S7 and S8). Because actin sequestering mechanism is highly unlikely at this low (1 nM) concentration, we hypothesized that latrunculin A might manifest its effects by capping the VopF/L-associated filaments. Because the drug prevents flattening of actin monomer at its pointed end required for the association with the barbed end (*55*), its incorporation at the barbed end is unlikely. Incorporation of latrunculin A (or rather latrunculin-bound actin protomers) at the pointed end is more plausible and, if favored by VopF/L, would lead to capping. Poisoning of VopF/L molecules that does not involve filament capping, e.g., due to a high-affinity binding of latrunculin/G-actin complexes to the toxins’ WH2 domains, is also possible. However, this scenario is less likely, as it would require multiple binding events to the six WH2 domains of the VopF or VopL homodimers, whose role in the processive elongation is redundant (fig. S5M). In contrast, barbed-end binding drug cytochalasin D, which potently suppresses the processive movement of the barbed-end actin elongator mDia1 (*38*), was effective against VopF/L only at much higher concentrations (1 μM; movie S6) likely acting by sequestering actin monomers.

To validate this unexpected finding, we aimed to affect actin elongation at a desired end by using polymerization-incompetent actin mutants with the mutations perturbing either barbed (DVD286,287,288AAA) (*37*) or pointed (AP204,243EK) (*56*) surface of actin monomers (Fig. 4, C to J). Because of the head-to-tail (barbed-to-pointed) nature of actin subunit interaction in the filament, the constructs with perturbed barbed surfaces can associate with the barbed end, preventing the incorporation of additional subunits there. The effects of such constructs on polymerization at the pointed end are minimal. Vice versa, the pointed surface mutants associate with and block the subsequent addition of WT actin subunits to the pointed end pausing the elongation; dissociation of the mutated actin subunits resumes the pointed-end elongation. Accordingly, impairing actin elongation in a filament end–specific manner using actin mutant with perturbed pointed surface resulted in a significantly reduced processivity of VopF speckles with shorter tracks and pauses, causing a decreased linearity of progression (Fig. 4, D to J). Those few VopF speckles still moving between the pauses in the presence of the pointed surface mutant had unchanged velocities (Fig. 4C). These effects were not observed in the presence of a barbed surface actin mutant, confirming the pointed-end selectivity of the effect. VopL speckles were affected by the actin mutants in a similar to VopF manner (movie S9).

Likewise, coexpression of VopF/L with either barbed-end capping protein (CP) (*57*, *58*) or pointed-end capping tropomodulin (Tmod-3) (*59*) revealed that CP did not affect the processive movement of VopF/L, while Tmod-3 had similar to the actin pointed surface mutant effects reducing the number of the processively moving speckles and significantly shortening their tracks (Fig. 4, K to R, and movie S9).

In contrast, the actin pointed surface mutant or Tmod-3 had no effect on the processive motility of the barbed-end actin polymerase mDia1, while the actin barbed surface mutant and CP interfered with it (fig. S6 and movie S10). High selectivity of the effects of the actin mutants, CP, and Tmod-3 on distinct actin polymerases (i.e., pointed-end VopF/L versus barbed-end mDia1) negates the possibility of large-scale nonspecific alterations on the actin cytoskeleton in the transfected cells. Accordingly, all the constructs caused no morphological or actin cytoskeleton aberrations when expressed in XTC cells at moderate to low levels used in this study (fig. S7). Together, these data strongly suggest that the observed intracellular processive movement of VopF/L stems from their ability to elongate actin filaments from their pointed ends.

### VopF processively elongates actin filaments in vitro

To assess minimal requirements for the motility of VopF/L, we used TIRFM in vitro analysis with purified protein components. We reasoned that the routinely used actin concentrations in TIRFM assays (~1 to 2 μM) may be insufficient to observe the pointed-end elongation of actin filaments. For comparison, the intracellular processivity of VopF/L occurs in an environment where G-actin concentration is typically at least an order of magnitude higher than what is used in traditional TIRFM experiments. To approach physiological conditions in a TIRFM chamber, we combined a higher concentration (10 μM) of unlabeled (to prevent otherwise overwhelmingly excessive fluorescence) actin in the presence of 12 μM PFN1 (to reduce spontaneous actin nucleation) and 20 nM CP (to inhibit actin polymerization at the barbed ends). We found that, under these conditions, the processive movement of fluorescently labeled SNAP-549-ΔNS-VopF molecules was supported by actin-PFN complexes (movie S11). An average track duration was ~1 min (61 ± 9.3 s), with some particles persistently moving for up to 174 s. SNAP-549-ΔNS-VopF velocity measured in vitro was ~2-fold lower than that of the corresponding EGFP-ΔNS-VopF construct in cellulo (93.4 ± 4.6 versus 182 ± 22 nm/s), tentatively reflecting ~2-fold higher concentrations of G-actin available for polymerization in the transfected cells, compared to the TIRF chamber.

**Fig. 5.**
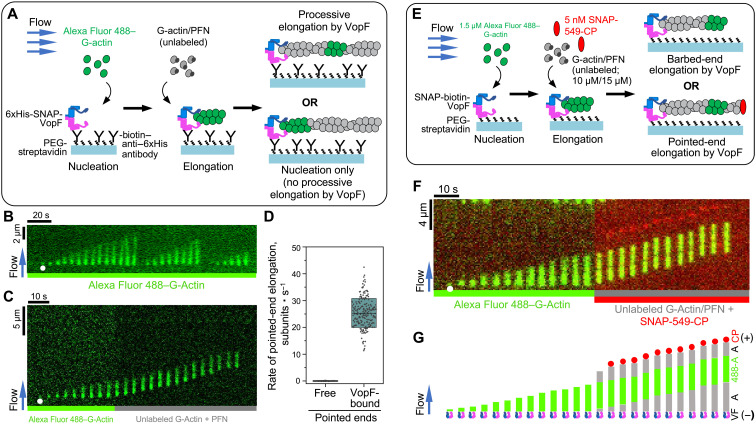
Direct visualization of processive pointed-end filament elongation by VopF using mf-TIRFM. (**A**) Schematic representation of the experimental strategy to test VopF’s processive filament elongation. Short actin filaments were nucleated by flowing in 1.5 μM Alexa Fluor 488–labeled actin monomers on the coverslip surface functionalized with 6xHis-SNAP-ΔNS-VopF molecules. These fluorescent filaments were then exposed to a flow containing 10 μM unlabeled actin monomers and 15 μM PFN. (**B**) An example kymograph showing repeated nucleation by the surface-anchored 6xHis-SNAP-ΔNS-VopF occurring at the same location (denoted by white circle) in the flow cell due to prolonged exposure to 1.5 μM Alexa Fluor 488–labeled actin monomers. (**C**) An example kymograph of a filament nucleated from labeled G-actin (1.5 μM Alexa Fluor 488–G-actin) by the surface-anchored 6xHis-SNAP-ΔNS-VopF and then elongating from PFN-bound unlabeled actin monomers (10 μM unlabeled actin monomers and 15 μM PFN). See also movie S12. (**D**) Elongation rates (means ± SD) of free (see movie S14) or VopF-assisted (see movie S12) pointed ends from 10 μM unlabeled actin monomers and 15 μM PFN. Number of filaments analyzed: 202, pooled from three independent experiments. (**E**) Schematic of the experimental strategy to identify the filament end of VopF’s processive elongation. (**F** and **G**) A kymograph derived from movie S13 (F) and a schematic (G) of a filament polymerizing from processive elongation by coverslip-bound biotin-ΔNS-VopF in an mf-TIRFM chamber. VF is SNAP-biotin-ΔNS-VopF, A is unlabeled actin, 488-A is Alexa Fluor 488–labeled actin, and CP is SNAP-549–labeled CP.

Because of the necessity to use nonfluorescent actin, VopF association with the ends is only inferred and not shown directly in the above experiments. To overcome this technical difficulty, we used mf-TIRF microscopy approach (*60*, *61*). Using glass coverslip-anchored 6xHis-tagged SNAP-ΔNS-VopF molecules (Fig. 5A), we observed surface-anchored filaments upon exposure to 1.5 μM Alexa Fluor 488–labeled actin monomers (Fig. 5B), in agreement with VopF’s known role in actin nucleation. Upon flowing 10 μM of unlabeled actin monomers in the presence of 15 μM PFN into the chamber, the preexisting fluorescent filament segments moved gradually at a rate of 25.3 ± 5.4 subunits/s (or 68.3 nm/s, assuming that one subunit produces a length increase of 2.7 nm) along the flow, suggesting that filament polymerization was occurring at the location of filament anchoring, i.e., at the site of coverslip-immobilized VopF (Fig. 5, C and D, and movie S12). The actin elongation rate in the mf-TIRF experiments is comparable to the rate of processively moving SNAP-549-ΔNS-VopF measured in the absence of flow in vitro (movie S11): 68.3 versus 93.4 nm/s. The small difference (1.37 times) is likely attributable to the surface-anchored versus free VopF in the mf-TIRF and conventional TIRFM experiments, respectively, and/or a possible variance in temperatures between the two experiments conducted in different laboratories. While these experiments demonstrate that VopF is a processive actin polymerase, they do not reveal whether VopF molecules are binding to the barbed or the pointed end of actin filaments.

### VopF is a processive pointed-end polymerase

To determine the polarity of filaments processively elongating from surface-anchored VopF in the mf-TIRF chamber, we included fluorescently labeled CP SNAP-549-CP as a barbed-end marker. Fluorescent filaments were nucleated from SNAP-biotin-ΔNS-VopF attached to the streptavidin-coated coverslip by flowing in 1.5 μM Alexa Fluor 488–labeled actin monomers. After the formation of short filaments was visually confirmed, the protein composition was switched to a mixture of 10 μM unlabeled actin with 15 μM PFN1 and 5 nM SNAP-549-CP (Fig. 5E). Notably, VopF-nucleated actin filaments continued to elongate at the ends associated with the VopF-functionalized glass surface (as evidenced by the appearance of a dark gap of unlabeled F-actin between VopF and Alexa Fluor 488–labeled F-actin segment) even when SNAP-549-CP was bound to the filament distal ends identifying them as barbed ends (Fig. 5, F and G, and movie S13). While the gap between the fluorescent portion of the filament and the point of anchoring (i.e., SNAP-biotin-ΔNS-VopF) was due to the VopF-assisted polymerization of the pointed end, the dark gap preceding SNAP-549-CP is due to the unassisted elongation of the barbed end before the capping event occurred (Fig. 5, F and G). In the control reaction without VopF, the elongation rate of free pointed ends from 10 μM actin and 15 μM PFN using actin filaments anchored by their barbed ends via CP was undetectable (Fig. 5D and movie S14).

Therefore, the in vitro reconstitution assays using only four proteins (actin, PFN, CP, and VopF) corroborate our cellular discovery that VopF is able to support the pointed-end elongation from actin-PFN complexes. Hence, VopF/L are bona fide processive pointed-end actin polymerases, which nucleate and elongate actin filaments while staying associated with their pointed ends.

### VopF/L disorganize cellular actin free barbed-end distribution

While the effects consistent with cell polarity disruption were reported for both VopF (*31*) and VopL (*28*) toxins, their mechanistic nature was not established. We hypothesized that uncontrollable actin pointed-end polymerization by VopF/L may disorganize actin barbed-end distribution, leading to the polarity disruption. Under physiological conditions, predominant localization of Arp2/3 complex activators at the cytolemma creates an intracellular gradient of barbed ends with their highest density at the leading edge (*62*). VopF/L-potentiated accumulation of F-actin may break this gradient and disrupt the highly orchestrated actin assembly process.

**Fig. 6.**
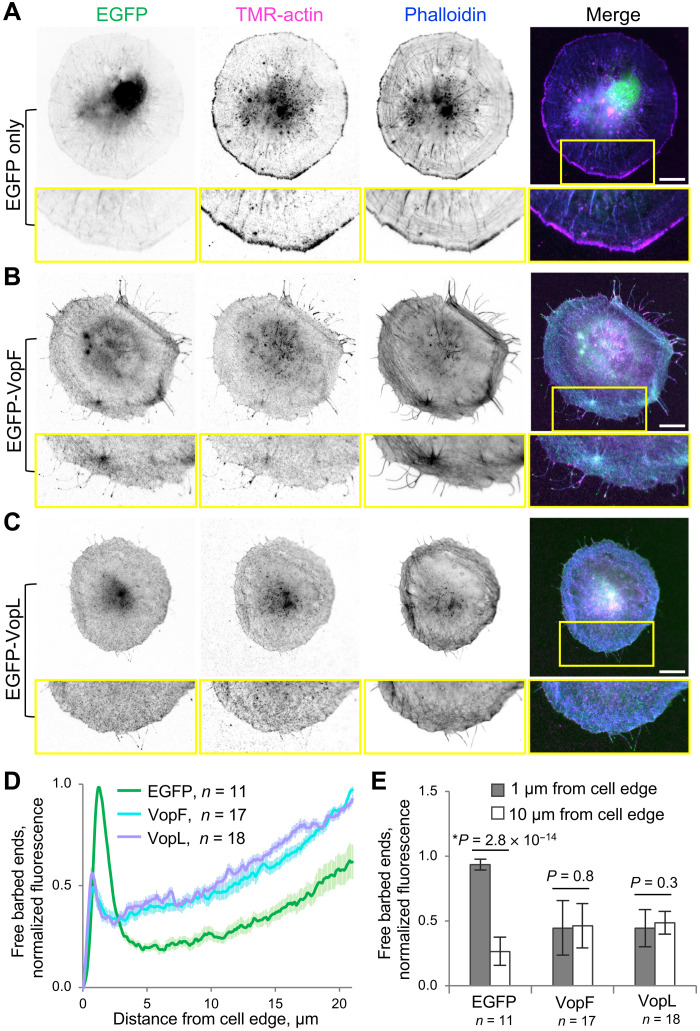
VopF and VopL disorganize actin free barbed-end distribution in transfected cells. (**A** to **C**) Representative images of transiently transfected spreading XTC cells expressing moderate levels of EGFP, EGFP-VopF, or EGFP-VopL. Cells were quickly fixed, permeabilized, and incubated with TMR-actin to label filaments’ free barbed ends and coumarin-phalloidin to label total F-actin. Zoomed views of the boxed areas are shown. Scale bars, 15 μm. (**D** and **E**) Normalized TMR-actin fluorescence intensity (i.e., labeled actin free barbed ends). Graphs show the fluorescence intensity plotted as a function of the distance from the cell edge (D) and a comparison between the fluorescence intensities at 1- and 10-μm distances from the cell edge (E). Numerical data are presented as means ± SE in (D) and means ± SD in (E); *n* is the number of cells.

Labeling of intracellular actin free barbed ends can be achieved by brief fixation/permeabilization followed by incubation of the cells with tetramethylrhodamine (TMR)–labeled actin (*62*, *63*). While the barbed ends were preferentially localized at the leading edge of the protruding lamellipodia in the EGFP only–expressing cells (Fig. 6A), the EGFP-VopF/L–expressing cells showed more uniform distribution of the barbed ends, which were not enriched at the cell edge (Fig. 6, B to E), confirming depolarization of the actin cytoskeleton. Furthermore, we analyzed the actin retrograde flow near the cell edge, which represents the lamellipodial actin dynamics under physiological condition, and found that it is mainly abrogated by VopF/L (movie S15). Therefore, the lamellipodium formation is disrupted by VopF/L, which corroborates our conclusion on the disruption of the actin cytoskeleton polarity.

## DISCUSSION

The classic paradigm of the actin cytoskeleton organization dictates that the actin filaments are assembled in precise cellular compartments via elongation at their barbed ends and disassembled primarily from their pointed ends. This functional polarity is supported and enhanced by host ABPs that act within this paradigm by (i) nucleating filaments only at the desired locations (e.g., at the inner cell and outer organelle membranes by the Arp2/3 complex and formins), (ii) promoting polymerization at the barbed end (e.g., PFN, formins, and Ena/VASP), (iii) fostering disassembly by filament severing and pointed end–specific depolymerization of aged filaments [e.g., ADF/cofilin, actin-interacting protein 1 (AIP1p), cyclase-associated protein (CAP1/2), and coronin], and (iv) blocking polymerization at the pointed end (by PFN). Apparent deviations from this trend appear to be unfavorable under cellular conditions. One exception to this is the recent report where twinfilin was shown to accelerate depolymerization of ADP-P_i_ barbed end under the assembly-promoting conditions in the presence of polymerizable actin monomers (*64*). For the above reasons, processive elongation of actin filaments at their pointed end is highly unfavorable under physiological conditions, is unexpected, and has not been observed before.

**Fig. 7.**
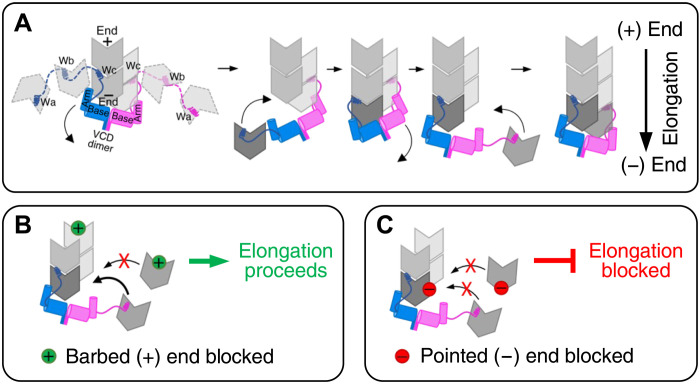
Schematic model of assisted pointed-end actin elongation by VopF/L. (**A**) VopF/L nucleate actin filaments from the pointed end. WH2 domains deliver new monomers, which are incorporated into the filament by a coordinated effort of VCD and WH2 domains. VopF/L stay associated with the filament pointed end during the repeated cycles of new monomer additions for the processive pointed-end actin elongation. (**B** and **C**) Schematics depicting the VopF/L-assisted elongation in the presence of actin barbed-end (B) and pointed-end (C) perturbations. (B) Barbed-end blocking agents (i.e., CP and CytD) and actin species with perturbed barbed-end surfaces cannot be added at the pointed end and therefore have little influence on the VopF/L-assisted pointed-end actin elongation. (C) Agents blocking actin pointed-ends (i.e., tropomodulin and LatA-bound actin monomers) as well as actin monomers with the perturbed pointed-end surface can get incorporated into the filament at the pointed end. In this case, however, the exposed blocked pointed-end surfaces prevent the addition of new subunits until the affected subunits are dissociated.

The detailed biochemical mechanism of the pointed-end processive polymerization by VopF/L calls for a separate full-scale investigation. However, the comparison of the data from our cellular experiments with the extensive in vitro data on the activity of VopF/L proteins published earlier (*32*, *33*, *36*, *37*) can provide some insight into the role of individual domains in actin nucleation and elongation (as schematically summarized in Fig. 7, A to C). Although bulk and classical TIRFM actin polymerization assays overlooked the processive elongation, they revealed and emphasized the nucleation events. On the other hand, nucleation events per se would be manifested as stationary, quickly dissociating speckles and can be easily missed by tracking the VopF/L dimers in live cells (unless nucleation is followed by processive elongation). A comparison of the results of the two approaches suggests that the VCD domain of VopF/L is not only essential but also insufficient for both nucleation and elongation, as in the absence of WH2 domains, it nucleates poorly (*36*) and fails to support the elongation (Fig. 3I). WH2 domains, on the other hand, differentially contribute to nucleation and elongation: While Wa seems to contribute strongly to nucleation (*33*), Wc is more important for elongation (Fig. 3P and fig. S5, L and M). PP-rich stretches are well recognized for recruiting PFN and actin-PFN complexes and are often found in proteins involved in actin polymerization at the barbed end. The presence of two PP regions in both VopF and VopL toxins (Fig. 1A) seemingly correlates with the toxins’ ability to use actin-PFN complexes as a substrate for processive filament elongation, except that the elongation at the pointed ends is hitherto unprecedented and would require dissociation of PFN before actin subunits can be incorporated. Furthermore, the Wc-VCD constructs missing both PP stretches are capable of elongation, albeit with reduced rates (Fig. 3, A, M, and P, and fig. S5, D and L), while deletion of the N-terminal PP region (PP1) has no effect on the VopF/L elongation rates (Fig. 3, A to C and N, and fig. S5, A, B, and K). Therefore, the ability of VopF/L toxins to incorporate actin at the pointed end using actin-PFN complexes as the substrate is among the most fascinating puzzles that remain to be resolved, as PFN plays a crucial role in establishing the polymerization polarity in vivo by blocking the pointed-end elongation.

Nucleation and processive elongation of the pointed end by VopF/L directly conflicts with the polarity-dictated paradigm of actin cytoskeleton organization and therefore may undermine the polarity of the cell by disrupting the polarity of the actin cytoskeleton. VopF/L can nucleate filaments independently of host factors (*32*) in any cytoplasmic compartment, while their ability to sustain a pointed-end elongation ensures that the filaments are sufficiently long to deplete the availability of G-actin for physiological needs. The reported mislocalization of tight junction protein ZO-1 in the presence of VopF (*31*) and NADPH (reduced form of nicotinamide adenine dinucleotide phosphate) oxidase complex in the presence of VopL (*28*) are, therefore, likely only two of the numerous yet to be discovered manifestations of this polarity-disrupting mechanism.

Is the polarity disruption via nucleation followed by pointed-end elongation more effective than either via the nucleation/dissociation cycles proposed originally for VopF/L proteins (*32*, *35*, *37*) or via a processive elongation of barbed ends? There are reasons to believe so. First, the sophisticated mechanisms of processive elongation require a coordinated engagement of several domains and therefore are unlikely to evolve and be retained without providing an evolutionary advantage to their producing organisms. The latter two mechanisms are likely to be less effective in depleting the G-actin content, as they would require effective competition with barbed-end binding CP, which is highly abundant, has subnanomolar affinity to actin, and is independent of the presence of tropomyosin. In contrast, the pointed-end capping Tmod is much less abundant, depends on the presence of tropomyosin, and, in its absence, has a notably lower affinity to the filament end. Because VopF/L nucleate filaments de novo, capping of the preexisting pointed ends (e.g., by Tmod or Arp2/3) should not affect the VopF/L processivity. Therefore, the pointed-end processive polymerases are expected to have less competition with cellular factors, which alone may justify their evolution. Moreover, one foreseeable consequence of the VopF/L-assisted processive filament elongation is that the F-actin pointed end will be enriched by adenosine triphosphate (ATP)– and ADP-P_i_–actin subunits, making it resistant to severing/depolymerization by ADF/cofilin and thus prolonging the effects of the disrupted polarity. If confirmed, this effect would indicate a compromised polarity at the individual filament level, in addition to the disruption of the conventional directionality of filament assemblies at the cellular level (as shown in Fig. 6).

Consistent with the cellular level polarity disruption mechanisms, VopF/L disrupt the lamellipodium as confirmed by an abolished actin retrograde flow (movie S15), which suggests a depletion of actin monomers available for physiological needs. The comparison of the actin polymerization rates measured in vitro (with known concentrations of the constituents) to those detected in living cells allows to estimate the actin concentration in the affected cells. Because the in vitro VopF/L-assisted polymerization rates at 10 μM actin-PFN complexes (93 nm/s with free VopF in movie S11 and ~68 nm/s in mf-TIRFM with the surface attached toxin in Fig. 5D) are ~2 to 2.6 times lower than the VopF/L motility/polymerization rates measured in the cell (~180 nm/s in Fig. 3), we can expect that the actin-PFN complex concentration in these cells should be ~20 to 25 μM. This value is substantially lower than the ~70 μM actin-PFN concentration proposed by Higashida *et al*. (*38*) based on the mDia1 propagation rates in the same cell line. These data corroborate the predicted lower G/F-actin ratio in the affected cells due to a higher F-actin content caused by the toxins.

Even under physiological conditions, distinct cellular actin networks compete for the same pool of G-actin (*65*). In the case of the VopF/L toxicity, the excessive formation of F-actin at abnormal locations inevitably shortens the supply for actin polymerization at the physiological sites, leading to a depletion of the filaments of normal polarity essential for cell needs. Moreover, the pointed-end processive elongation causes other intriguing phenomena of compromised polarity, the significance of which for the pathogenesis remains to be established. Specifically, within the VopF/L-induced filopodia-like protrusions observed in the affected cells, the toxins move persistently toward the distal ends, suggesting that at least some actin filaments should have polarity opposite to that in conventional filopodia. It would be interesting to check whether such VopF/L-induced protrusions may serve as traps for proteins that are originally destined to be translocated from the tips of normal filopodia to the cytoplasm (i.e., in the direction from the barbed toward pointed ends of actin filaments).

Bacterial toxins have become invaluable tools for understanding intricate aspects of the actin cytoskeleton and the cell at large. VopF/L toxins will undoubtedly continue this trend. While it remains to be established whether any of the host proteins have the ability similar to VopF/L to elongate actin from the pointed end, the present study demonstrates that the actin filament design allows for this possibility.

## MATERIALS AND METHODS

### Cell culture

U2OS cells used in the initial experiments to determine overall cellular effects of the toxins (Fig. 1 and movie S1) were cultured in Dulbecco’s modified Eagle’s medium (Corning, Corning, NY) supplemented with 1% penicillin-streptomycin (Cytiva/HyClone, Marlborough, MA) and 10% fetal bovine serum (Corning) at 37°C with 5% CO_2_ in a humidified incubator. The identity and purity of the U2OS cells were verified by short tandem repeat profiling (amelogenin + nine loci) at the Genomics Shared Resource (Ohio State University Comprehensive Cancer Center, Columbus, OH). XTC cells (a gift from N. Watanabe), which were used for comparison of cellular effects (figs. S1, S3, A and B, and S7), for actin free barbed-end distribution analysis (Fig. 6), and for all single-molecule live-cell experiments, were cultured in 70% Leibovitz’s L-15 medium (HiMedia, Chester, PA) supplemented with 1% penicillin-streptomycin and 10% fetal bovine serum at 23°C without CO_2_ equilibration. Both cell lines were mycoplasma negative as determined by polymerase chain reaction (PCR) tests.

### Transient transfections and DNA constructs

Transfections of U2OS and XTC cells were performed using Lipofectamine 3000 transfection reagent (Thermo Fisher Scientific, Waltham, MA) according to the manufacturer’s instructions. For cloning, cDNA products were amplified using Q5 High-Fidelity DNA Polymerase (New England Biolabs, Ipswich, MA) and inserted into corresponding vectors using NEBuilder HiFi DNA Assembly Master Mix (New England Biolabs, Ipswich, MA) followed by transformation into in-house prepared Z-competent XL10-Gold *Escherichia coli* cells (Agilent Technologies, Santa Clara, CA) using the Mix & Go! *E. coli* Transformation Kit (Zymo Research, Irvine, CA). Plasmid DNA was purified using Zyppy Plasmid Miniprep and ZymoPURE II Midiprep kits (Zymo Research, Irvine, CA). The following constructs were used for transfections: VopF and VopL constructs (shown in Fig. 3A) were generated by amplifying *VopF* cDNA (from pBG1805-VopF; a gift from M. Dziejman) and *VopL* cDNA (from pSFFV-VopL; a gift from K. Orth) (*28*) and inserting the amplified cDNAs into dCMV-EGFP plasmid as was previously reported for other proteins (*45*). Mouse CP was subcloned as heterodimer (*CapZ*α*1*/*CapZ*β*2*) from pRSF-Duet-CP plasmid (a gift from J. Cooper) (*66*) into a polycistronic vector with T2A “self-cleaving” peptide and mCherry inserted in-frame between the CapZα1 and CapZβ2 subunits. Mouse *Tmod-3* was subcloned from pCMV5-HA-mTmod3 [RRID:Addgene_70752 (*67*)] into pmCherry vector (Takara Bio USA, San Jose, CA). dCMV-EGFP-mDia1-ΔN3 (*38*) and dCMV-EGFP-mDia2-ΔN3 were obtained from N. Watanabe. pEGFP (Clontech/Takara Bio USA, San Jose, CA) and pmCherry (Takara Bio USA, San Jose, CA) vectors were used as controls. EB3-tdTomato [RRID:Addgene_50708 (*68*)] was used as a marker of microtubule growth. pmCherry-Utr-CH [RRID:Addgene_26740 (*69*)] expressing actin-binding domain of utrophin and pmCherry-F-tractin [RRID:Addgene_155218 (*70*)] were used as F-actin markers. pmCherry-β-actin (RRID:Addgene_54966) was used to generate barbed and pointed surface actin mutants carrying DVD286,287,288AAA (*37*) and AP204,243EK (*56*) mutations, respectively. Mutagenesis was performed using the QuikChange Site-Directed Mutagenesis Kit (Agilent Technologies, Santa Clara, CA). dCMV-TagRFPT-actin plasmid was generated by subcloning of β-actin in-frame with an N-terminal TagRFPT tag into dCMV vector.

### Protein purification and fluorescent labeling

Skeletal actin was prepared from acetone powder of rabbit skeletal muscle (Pel-Freez Biologicals, Rogers, AR) as previously described using G-buffer (5 mM tris-HCl, 0.2 mM CaCl_2_, 0.2 mM ATP, and 5 mM β-mercaptoethanol) and multiple rounds of polymerization and depolymerization (*71*). Alexa Fluor 488– and TMR (Molecular Probes/Thermo Fisher Scientific, Waltham, MA)–labeled actin samples were prepared from G-actin in G-buffer devoid of reducing agents as previously described (*45*). *N*-(1-pyrenyl)iodoacetamide (pyrene; Thermo Fisher Scientific, Waltham, MA)–labeled actin was prepared from F-actin followed by depolymerization upon dialysis in G-buffer as described (*72*, *73*). Labeled and unlabeled G-actin was further purified by size exclusion chromatography (Sephacryl S200-HR; Cytiva/GE Healthcare, Marlborough, MA). Unlabeled actin was stored on ice in G-buffer containing β-mercaptoethanol and used within 4 weeks with a dialysis to G-buffer after 2 weeks of storage. Labeled G-actin was snap-frozen and stored at −80°C.

Recombinant proteins were expressed in BL21-CodonPlus(DE3)-RP *E. coli* (Agilent Technologies, Santa Clara, CA) unless indicated otherwise. Human PFN1 was purified as previously described (*74*). PFN1 was bound to a poly-l-proline sepharose resin, eluted under denaturing conditions, and dialyzed against three buffer changes of storage buffer [2 mM tris-HCl (pH 8.0), 0.2 mM EGTA, 1 mM dithiothreitol (DTT), and 0.1 mM phenylmethylsulfonyl fluoride (PMSF)].

Δ*NS-VopF* was subcloned downstream of the *SNAP26b* gene in pSNAP-tag(T7)-2 vector (New England Biolabs) in-frame with 6xHis-tag using Q5 High-Fidelity DNA Polymerase (New England Biolabs) to amplify cDNA and NEBuilder HiFi DNA Assembly Master Mix (New England Biolabs, Ipswich, MA). 6xHis-SNAP-tagged ΔNS-VopF was purified using TALON Metal Affinity Resin (Takara Bio USA, San Jose, CA) according to the manufacturer’s instructions. For labeling, SNAP-Surface-549 or SNAP-biotin (New England Biolabs, Ipswich, MA) were added at 2-mole excess in a buffer containing 20 mM Hepes (pH 7.5), 150 mM NaCl, 1 mM DTT, and 0.1 mM PMSF overnight at 4°C, followed by gel filtration or dialysis to remove free dye.

His-tagged mouse CP heterodimer (α1 and β2 subunits in pRSFDuet, a gift from J. Cooper) was purified as described previously (*66*) using TALON Metal Affinity Resin (Takara Bio USA, San Jose, CA) followed by a ceramic hydroxyapatite column (40 μm; EconoFit CHT type I, Bio-Rad Laboratories, Hercules, CA) and gel filtration (Sephacryl S-200 HR, Cytiva/GE Healthcare, Marlborough, MA). The purified CP was stored in 20 mM Mops, 100 mM KCl, and 1 mM tris(2-carboxyethyl)phosphine (TCEP) (pH 7.2) at −80°C.

SNAP-tagged CP was expressed in *E. coli* BL21 DE3 (Gold Biotechnology Inc., Olivette, MO) by growing cells to log phase at 37°C in terrific broth medium and then inducing expression using 1 mM isopropyl-β-d-thiogalactopyranoside (IPTG) at 18°C overnight. Cells were harvested by centrifugation, and pellets were stored at −80°C. Frozen pellets were resuspended in lysis buffer [20 mM NaPO_4_ (pH 7.8), 300 mM NaCl, 1 mM DTT, 15 mM imidazole, and 1 mM PMSF] supplemented with a protease inhibitor cocktail (0.5 μM each of pepstatin A, antipain, leupeptin, aprotinin, and chymostatin). Cells were lysed by sonication with a tip sonicator while keeping the tubes on ice. The lysate was cleared by centrifugation at 150,000*g* for 30 min at 4°C. The supernatant was incubated with Ni–nitrilotriacetic acid agarose beads (Qiagen, Hilden, Germany) for 2 hours at 4°C, followed by extensive washing of the beads with washing buffer [20 mM NaPO_4_ (pH 7.8), 300 mM NaCl, 1 mM DTT, and 25 mM imidazole] to remove nonspecifically bound proteins. SNAP-CP was eluted using 20 mM NaPO_4_ (pH 7.8), 300 mM NaCl, 250 mM imidazole, and 1 mM DTT. The eluted protein was concentrated and labeled with SNAP-Surface 549 or SNAP-biotin (New England Biolabs, Ipswich, MA) according to the manufacturer’s instructions. Free dye was removed using size exclusion chromatography by loading the labeled protein on a Superdex 200 Increase 10/300 GL gel filtration column (Cytiva/GE Healthcare, Marlborough, MA) eluted with 20 mM Hepes (pH 7.5), 150 mM KCl, and 0.5 mM DTT. Fractions containing the protein were combined. Protein concentration and labeling fraction were determined by measuring the absorbance at 280 and 560 nm (ε_280_ = 102,165 M^−1^ cm^−1^ and ε_560_ = 140,300 M^−1^ cm^−1^). Purified protein was aliquoted, snap-frozen in liquid N_2_, and stored at −80°C.

Protocatechuate 3,4-dioxygenase (PCD) from *Pseudomonas putida* was expressed from pVP91A-pcaHG plasmid [RRID:Addgene_113766 (*75*)] in Rosetta(DE3) *E. coli* cells (MilliporeSigma, Burlington, MA) in LB media supplemented with 0.5 mM MgSO_4_ and 1 mM CaCl_2_. Protein expression was induced by addition of 250 μM IPTG, and the culture was supplemented with 25 μM ferrous ammonium sulfate and grown overnight at 15°C. Cells were resuspended in 50 mM sodium phosphate buffer (pH 8.0) containing 150 mM NaCl, 25 μM ferrous ammonium sulfate, and 1 mM PMSF and lysed by French press. PCD was purified using TALON Metal Affinity Resin (Takara Bio USA, San Jose, CA) followed by size exclusion chromatography. Purified PCD was stored in 50% glycerol, 10 mM Hepes (pH 8.0), 75 mM NaCl, and 0.05 mM PMSF at −80°C.

### Intracellular F-actin content analysis

U2OS cells transfected with mCherry were mixed with U2OS cells transfected with EGFP, EGFP-VopF, or EGFP-VopL. The mixed cell populations were fixed in 4% paraformaldehyde, permeabilized with 0.1% of Triton X-100 in phosphate-buffered saline (pH 7.4), stained with coumarin-phalloidin (Santa Cruz Biotechnology, Dallas, TX), and imaged using a Nikon Eclipse Ti-E microscope equipped with a Nikon DS-Qi1Mc camera (Nikon Instruments, Melville, NY) under identical conditions. Each image included experimental EGFP-positive cells and mCherry-positive cells, where latter served as an internal negative control. Differentially transfected (mCherry-positive and EGFP-positive) cells expressing moderate amount of a fluorescently tagged protein of interest were manually outlined to measure the integrated density of coumarin-phalloidin and cell area in Fiji (ImageJ) software (*42*). Ratios of the integrated densities to the cell areas were calculated for each cell and corrected for a background fluorescence. The obtained mean fluorescence intensities for each cell were averaged between the cell groups (mCherry-positive and EGFP-positive) and normalized to the value of the control mCherry-positive cells (individual cell numbers for each transfection construct are indicated in Fig. 1C).

### Actin free barbed-end distribution analysis

Actin free barbed ends were labeled as described previously (*62*, *63*) with some modifications. XTC cells transfected with EGFP, EGFP-VopF, or EGFP-VopL were seeded on poly-d-lysine coverslips (Neuvitro Corporation, Vancouver, WA) coated with 3 μM concanavalin A in 10 mM MES (pH 6.1). After 30-min spreading in L-15 medium without fetal bovine serum, cells were rapidly fixed with 4% paraformaldehyde in cytoskeleton buffer [10 mM MES (pH 6.1), 138 mM KCl, 3 mM MgCl_2_, and 2 mM EGTA] for 30 s and carefully washed with rinsing buffer [20 mM Hepes (pH 7.5), 138 mM KCl, 4 mM MgCl_2_, and 3 mM EGTA] supplemented with saponin (0.25 mg/ml). Cells were incubated for 2 min in the rinsing buffer containing coumarin-phalloidin (0.5 μg/ml), 1 mM ATP, saponin (0.25 mg/ml), and 1 μM TMR-labeled actin monomers. TMR-actin was pre-spun at 300,000*g* for 30 min at 4°C and added to the mixture immediately before use. Following labeling, cells were washed with the rinsing buffer, fixed with 4% paraformaldehyde in the cytoskeleton buffer for 10 min, washed in the cytoskeleton buffer, mounted in 10 mM tris-HCl (pH 8.5) containing 70% glycerol and 1% 1,4-diazabicyclo[2.2.2]octane (DABCO), and imaged using a Nikon Eclipse Ti-E microscope equipped with a Nikon CFI Plan Apochromat λ 60× oil objective [numerical aperture (NA): 1.40] and a Nikon DS-Qi1Mc camera (Nikon Instruments, Melville, NY). TMR-actin distribution along a cross section perpendicular to the leading edge was measured in individual cells expressing moderate amount of a fluorescently tagged protein of interest; 10 line profiles were averaged per cell (individual cell numbers for each transfection construct are indicated in Fig. 6, D and E). Fluorescence intensity was normalized to the highest signal in each cell set as 1, and the background signal was set as 0.

### TIRF SiMS live-cell microscopy

For SiMS analysis (Figs. 2 to 4, figs. S2 and S4 to S6, and movies S3 to S10 and S15), transfected XTC cells were seeded onto poly-d-lysine–coated coverslips in Attofluor chambers (Thermo Fisher Scientific, Waltham, MA) and imaged using TIRF module (15-mW laser) on a Nikon Eclipse Ti-E inverted microscope equipped with perfect focus system, a Nikon CFI Plan Apochromat λ 100× oil objective (NA 1.45), and an iXon Ultra 897 EMCCD (electron-multiplying charge-coupled device) camera (Andor Technology, Belfast, UK). A field diaphragm was used to restrict the illumination (10% of laser power) to a small area at the cell edge to minimize cell photodamage. Cells expressing very low levels (suitable for SiMS analysis) of EGFP-tagged proteins under the dCMV promoter (*41*) were identified and selected for imaging. For double transfections (Fig. 4 and movies S9 and S10), cells expressing very low levels of EGFP-tagged toxins and moderate to low expression levels of either actin mutants, CP, or Tmod-3 (such as those shown in fig. S7) were used. Time-lapse images were taken for the duration of 2 min with 1-s (VopF), 2-s (VopL), or 0.25-s (mDia1-ΔN3) intervals. For experiments using inhibitors, time-lapse images were taken before and after the addition of the indicated drugs: jasplakinolide (MilliporeSigma, Burlington, MA), swinholide A (AdipoGen, San Diego, CA), cytochalasin D (Enzo Life Sciences, Farmingdale, NY), latrunculin A (Enzo Life Sciences, Farmingdale, NY), microtubule inhibitor nocodazole (Thermo Fisher Scientific, Waltham, MA), and myosin inhibitors blebbistatin (APExBIO, Houston, TX) and MyoVin1 (MilliporeSigma, Burlington, MA). Average and maximum intensity projections and kymographs of the time-lapse images were obtained using Fiji Z-project tool and KymographBuilder plug-in, respectively. Movie montages were assembled using Multi Stack Montage Fiji plug-in.

### Single-molecule tracking analysis

Single-particle tracking was performed using Fiji plug-in TrackMate (*43*). To identify processively moving speckles, the following two track filters were applied (as described in fig. S2):

1) “Track duration” filter: Processively moving speckles can be identified if they appear for at least three consecutive frames, which represent 6 s (at a rate of one frame per 2 s in the case of VopL). Because VopF speckles move ~2 times faster than VopL, VopF time-lapse images were acquired at a rate of one frame per second. However, for consistency, both VopL and VopF speckles were treated with the same track duration filter of at least 6 s.

2) “Confinement ratio (CR)” filter (i.e., persistence defined as the net displacement divided by the total distance traveled) was applied to separate processively moving tracks (CR > 0.5) from those of stationary or randomly moving speckles (CR < 0.5).

Kymograph analysis was performed using Fiji Measure tool (bounding rectangle option): Velocities were calculated by dividing the traveled distance (*d*) by the time (*t*) using the bounding rectangle for the line drawn to trace the speckle tracks on the kymographs. Individual numbers of cells quantified by TrackMate and kymograph analyses are given in Fig. 2.

### Stepwise photobleaching analysis

To determine oligomerization state, fluorescence intensities of VopF/L intracellular speckles were monitored upon photobleaching. Transfected XTC cells were seeded onto poly-d-lysine coverslips. Cells expressing very low levels of EGFP-VopF or EGFP-VopL (suitable for SiMS analysis) were identified and selected for imaging. VopF and VopL speckle dynamics was arrested by addition of latrunculin A (at 10 nM final concentration). TIRFM images were acquired at a rate of two frames per second with continuous illumination using a 488-nm laser to excite EGFP. Background fluorescence was subtracted using Fiji background subtraction tool with a rolling ball radius set to five pixels. Speckles were manually identified, and their fluorescence intensity profiles over time were obtained using Fiji ROI manager tool. Stepwise reductions in fluorescence intensity profiles were subjectively determined and counted manually. In cases where more than one EGFP molecules were bleached simultaneously and individual steps were not resolved, the number of steps was deduced on the basis of the initial fluorescence level. To determine EGFP-VopF and EGFP-VopL oligomerization states, the distribution of the photobleaching steps was compared to a binomial distribution model as described previously (*76*), where probability of EGFP being fluorescent (s) is 0.75, which is in agreement with the previously published data on EGFP photobleaching analysis (*77*). The individual number of cells and speckles quantified is given in fig. S4.

### Conventional in vitro TIRFM reconstituted assays

For in vitro reconstituted TIRFM experiments (movie S11), Ca^2+^-ATP G-actin (final concentration, 10 μM; 33% Alexa Fluor 488–labeled) was switched to Mg^2+^-ATP G-actin by a 2-min incubation in 0.05 mM MgCl_2_ and 0.2 mM EGTA. PCD/protocatechuic acid (PCA) oxygen scavenging system (*78*) was used. Actin was mixed with 20 nM unlabeled CP and 4 nM SNAP-549-ΔNS-VopF in the presence of 12 μM PFN1 in the final reaction buffer (pH 7.0): 10 mM imidazole, 0.2 mM EGTA, 1 mM MgCl_2_, 50 mM KCl, 0.25 mM ATP, 10 mM ascorbic acid, 2.5 mM PCA (MilliporeSigma, Burlington, MA), 0.1% bovine serum albumin (VWR International, Radnor, PA), 0.6% methylcellulose-400cP (MilliporeSigma, Burlington, MA), and 0.1 μM PCD. Immediately upon mixing, reactions were transferred to a TIRF chamber and imaged using a Nikon Eclipse Ti-E microscope equipped with a TIRF illumination module (with a 15-mW laser), a Nikon CFI Plan Apochromat λ 100× oil objective (NA: 1.45), a perfect focus system (Nikon Instruments, Melville, NY), and an iXon Ultra 897 EMCCD camera (Andor Technology, Belfast, UK).

### mf-TIRFM assays

For in vitro mf-TIRF experiments (*61*), glass coverslips were first cleaned via successive sonications in 2% Micro 90 detergent, 1 M HCl, 1 M KOH, and 200 proof ethanol. Cleaned coverslips were then dried under an N_2_ stream and coated with 80% ethanol solution (pH 2.0) containing polyethylene glycol (PEG)–silane (2 mg/ml) and PEG-biotin-silane (50 μg/ml; Laysan Bio, Arab, AL). The coated coverslips were then incubated overnight in an oven at 70°C. On the day of imaging, coverslips were rinsed with H_2_O and dried with N_2_ before attaching to a polydimethylsiloxane chamber with one outlet and three inlets. This assembled flow cell was then connected to a MAESFLO microfluidic flow control system (Fluigent, France). The chamber was then rinsed with TIRF buffer [10 mM imidazole (pH 7.4), 50 mM KCl, 1 mM MgCl_2_, 1 mM EGTA, 0.2 mM ATP, 1 mM DABCO, and 25 mM DTT] and incubated with 1% bovine serum albumin containing streptavidin (10 μg/ml) for 5 min. Two distinct approaches were used for surface anchoring of VopF molecules. In the first strategy (Fig. 5, A to C, and movie S12), the chamber was sequentially incubated with biotinylated anti–6xHis-tag antibody (RRID:AB_2536983; Thermo Fisher Scientific, Waltham, MA) and 6xHis-tagged SNAP-ΔNS-VopF. In the second strategy (Fig. 5, E to G, and movie S13), streptavidin-coated coverslips were directly exposed to SNAP-biotin-ΔNS-VopF. After a 5-min wash with TIRF buffer to remove unbound protein, actin filaments were nucleated from surface-bound VopF molecules by flowing in a solution containing 1.5 μM Alexa Fluor 488–labeled actin monomers (in TIRF buffer) and elongated by flowing in a solution containing 10 μM unlabeled actin monomers with 15 μM PFN1 (with or without 5 nM SNAP-549-CP).

To determine the rate of unassisted elongation of free pointed ends (Fig. 5D and movie S14), fluorescently labeled preformed filaments were captured on the coverslip surface by coverslip-anchored biotinylated SNAP-CP. The filaments were exposed to alternating flows of 10 μM Alexa Fluor 488–G-actin with 15 μM PFN (for 55 s of every minute) and 10 μM unlabeled G-actin with 15 μM PFN (for 5 s every min). TIRFM images were acquired once every minute during the period when unlabeled actin was flowing. To prevent the surface attachment of spontaneously nucleated filaments from the flow, 1 μM free CP (nonbiotinylated) was added to both labeled and unlabeled actin/PFN mixtures.

All experiments were carried out at room temperature in TIRF buffer. Each experiment was repeated at least three times and yielded similar results. Single- and multi-wavelength time-lapse TIRF imaging were performed using a Nikon Ti2-E inverted microscope equipped with a 20-mW laser and a 60× TIRF objective with an NA of 1.49 (Nikon Instruments Inc.) and an EMCCD camera (Andor iXon). One pixel was equivalent to 143 × 143 nm. During measurements, optimal focus was maintained by the Perfect Focus system (Nikon Instruments, Melville, NY). Images were corrected for background fluorescence using the Fiji rolling ball background subtraction algorithm (ball radius: 25 pixels) and analyzed using the Fiji kymograph plug-in. The kymograph slopes were measured to determine rates of polymerization for individual filaments. One actin subunit was taken to contribute 2.7 nm to filament length.

### Bulk pyrenyl-actin polymerization assays

Bulk pyrenyl-actin polymerization assays (fig. S3C) were conducted as described (*45*). Briefly, 2.5 μM (5% pyrene-labeled) G-actin was premixed with 3 μM (final concentration) of actin-targeting drugs for 5 min. The reactions in 96-well half-area black nonbinding plates (Corning, Corning, NY) were transferred to a Tecan M1000 Pro plate reader (Tecan US Inc., Morrisville, NC), and pyrene fluorescence was monitored with λ_ex_ of 365 nm and λ_em_ of 407 nm. Actin was switched from Ca^2+^-bound to Mg^2+^-bound state by adding EGTA and MgCl_2_ to final concentrations of 0.5 and 1 mM, respectively. Following a 1- to 2-min incubation, 4× initiation buffer [40 mM Mops (pH 7.0), 0.8 mM ATP, 2 mM DTT, 4 mM MgCl_2_, and 200 mM KCl] was added to a final concentration of 1×, and the changes in fluorescence signal were continued to be recorded.

### Statistical analysis

Statistical significance was determined using Analysis ToolPak (a Microsoft Excel add-in): For two-sample comparisons, an unpaired two-tailed Student’s *t* test was used (Figs. 1C, 2, E and F, 4, I, J, Q, and R, and 6E and fig. S2, E to I); for multiple comparisons, a one-way analysis of variance (ANOVA) followed by multiple comparison tests with Bonferroni correction was applied (Figs. 2D, 3, N to P, and 4, C to F and K to N, and figs. S5, K to M, and S6C). No data were excluded from the analyses. For live-cell TIRFM, only the cells with low to moderate expression levels of the transfected proteins that were well spread within 30 min after plating were used. Following this procedure ensured that the actin cytoskeleton in the analyzed cells was sufficiently preserved to allow the spreading. All statistical analyses were performed on samples pooled from at least three independent experiments, numerical data are presented as mean values, and error bars represent SD or SEM as indicated in the corresponding figure legends. The details of the statistical analysis, including individual *P* values, values of *n*, and error bar definitions, are included in the figures and figure legends. All representative micrographs were selected from datasets obtained from at least three independent experiments.

## Supplementary Materials

### This PDF file includes:

Figs. S1 to S7 References
