# Supplementary material for: Pointed-end processive elongation of actin filaments by Vibrio effectors VopF and VopL
Source: Sci Adv. 2022 Nov 18;8(46):eadc9239. doi: 10.1126/sciadv.adc9239 (PMC9674292; doi:10.1126/sciadv.adc9239)
Supplement: Supplementary file 1 — Figs. S1 to S7 References [file sciadv.adc9239_sm.pdf]

Science Advances
AAAS

Supplementary Materials for

Pointed-end processive elongation of actin filaments by *Vibrio* effectors VopF and VopL

Elena Kudryashova *et al.*

Corresponding author: Elena Kudryashova, kudryashova.1@osu.edu;
Dmitri S. Kudryashov, kudryashov.1@osu.edu

Sci. Adv. **8**, eadc9239 (2022)
DOI: 10.1126/sciadv.adc9239

The PDF file includes:

Figs. S1 to S7
Legends for movies S1 to S15
References

Other Supplementary Material for this manuscript includes the following:

Movies S1 to S15

Figures S1 to S7

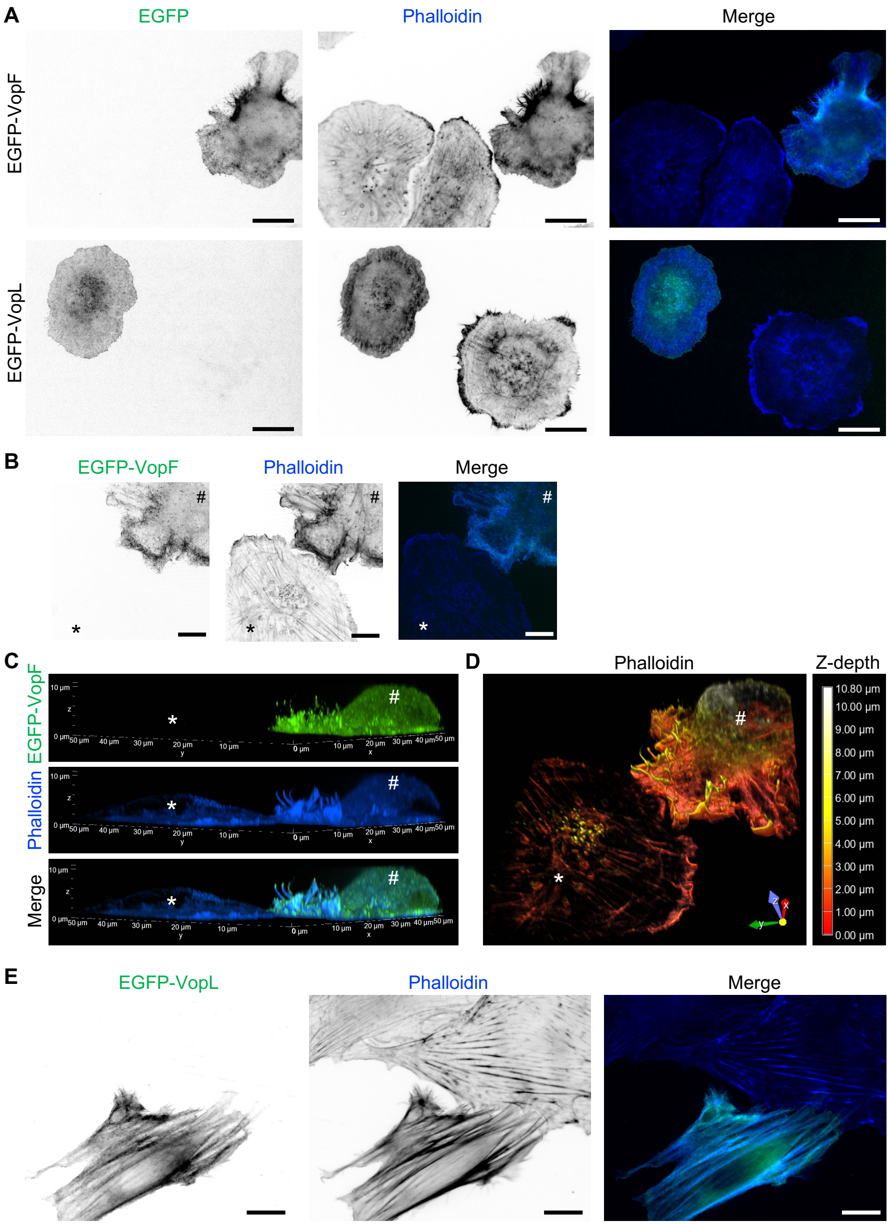

Fig. S1. VopF and VopL induce excessive accumulation of F-actin and formation of thin protrusions in transfected cells. (**A**) Representative images of transiently transfected spreading XTC cells (30 min post-plating) expressing moderate levels of EGFP-tagged VopF or VopL and contra stained with coumarin-phalloidin to label F-actin were obtained using TIRFM. Scale bars are 20 µm. (**B**-**D**) Maximum intensity projections (MIP) of Z-stack images of spreading XTC cells transiently transfected with EGFP-VopF and contra stained with coumarin-phalloidin obtained using Spinning Disk Super Resolution CSU-W1 SoRa (Yokogawa Life Sciences/Nikon). # denotes EGFP-VopF expressing cell; * indicates non-transfected control cell. (**B**) 2D MIP Z-stack images; scale bars are 10 µm. Side view of 3D MIP Z-stacks (**C**) and depth-coded 3D MIP of the phallodin channel Z-stack (**D**) of the same image shown in B. (**E**) TIRFM images of 24 h post-plating XTC cells transfected with EGFP-VopL and contra stained with coumarin-phalloidin. Scale bars are 20 µm.

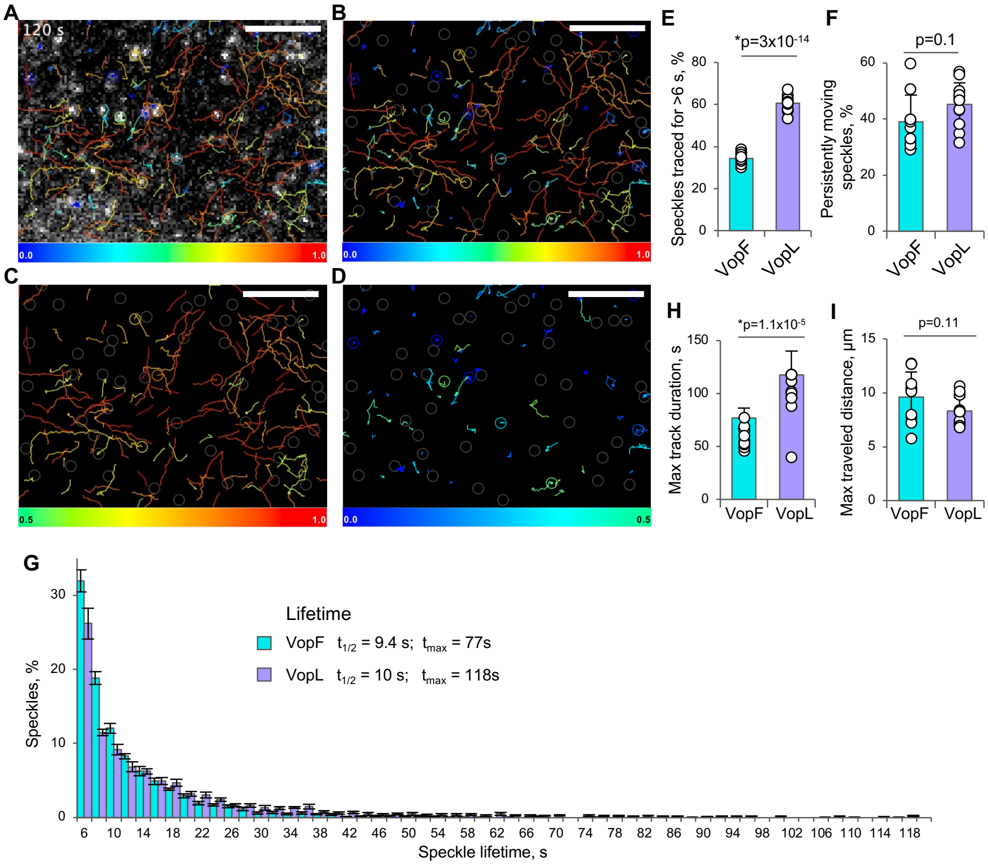

Fig. S2. TrackMate analysis of the persistently moving VopF and VopL speckles. Transiently transfected spreading XTC cells expressing very low (*i.e.*, suitable for single-molecule analysis) levels of EGFP-tagged VopF or VopL were selected for TIRF SiMS imaging. (**A-D**) TrackMate analysis of TIRF SiMS images shows the trajectories of EGFP-VopF speckles at a final time frame of 120 sec (see also movie S4); the image in **A** includes the actual image of speckles in addition to the trajectories (**B-D**). Individual speckles are shown as circles (spots) at each time frame (they do not accumulate with time). Automatically tracked speckle trajectories are cumulative, and the last frame (shown) contains all the identified tracks. The tracks and the corresponding spots are colored according to the confinement ratios (CR) from 0 to 1 (color schemes are shown below each image). Images in **A** and **B** show all tracks after applying the track duration filter (at least three consecutive frames, *i.e.*, > 6 sec); Images in **C** and **D** show tracks after applying the additional confinement ratio filter (CR < 0.5 or CR > 0.5 for **C** and **D**, respectively). Grey circles in **A** and **B** are unclassified speckles (tracked for <6 s, *i.e.*, filtered out with the track duration filter). In **C** and **D**, grey circles include both types of circles, unclassified and filtered out with the corresponding confinement ratio filter. Scale bars are 5 µm. (**E-I**) Graphs show percent of speckles (mean ± SD) traced for more than 6 s (**E**), percent of speckles (mean ± SD) moving processively (*i.e.*, CR > 0.5) out of the speckles persisted for > 6 s (**F**), lifetime distribution (mean ± SEM) of the processively moving speckles (histogram; **G**), maximum track duration (mean ± SD (**H**)), and maximum track length (mean ± SD (**I**)); n=10 cells (144-814 tracks per cell; total 3501) for VopF; n=12 cells (114-714 tracks per cell; total 3357) for VopL.

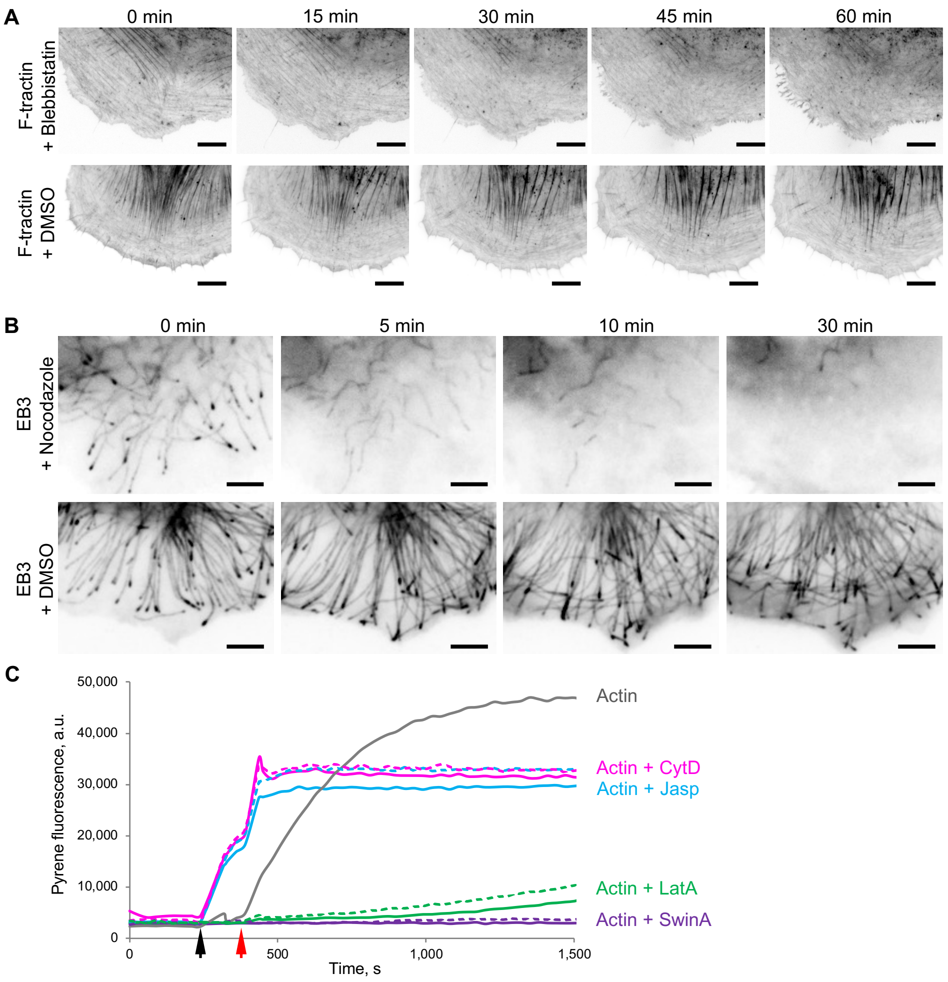

Fig. S3. Activity of the actin, myosin, and microtubule inhibitors used in this study. (**A**) As expected, blebbistatin (*79*) induces characteristic disruption of stress fibers and loss of cell adhesion, in comparison with vehicle-treated (DMSO) control cells. Representative time-lapse images of XTC cells transfected with mCherry-F-tractin (an F-actin marker) and treated with 25 µM blebbistatin (or DMSO) are shown. Scale bars are 10 µm. (**B**) Nocodazole treatment of XTC cells disrupts microtubules, in comparison with vehicle-treated (DMSO) control cells. Representative time-lapse images of XTC cells transfected with EB3-tdTomato (a microtubule plus-end binding protein) and treated with 10 µM nocodazole (or DMSO) are shown. Scale bars are 5 µm. (**C**) Bulk pyrenyl-actin polymerization assays using 2.5 µM actin (5% pyrene-labeled). In accordance with the respective mechanisms of action, jasplakinolide [Jasp; (*48*, *49*)] increases the pyrenyl-actin fluorescence by potentiating actin polymerization; cytochalasin D [CytD; (*52*, *80*)] increases the initial rate of pyrene fluorescence consistent with the previously reported effects of Mg^{2+}-dependent formation of CytD-induced dimers (*80*); both, swinholide A [SwinA; (*50*, *81*)] and latrunculin A [LatA; (*53*, *55*)] have an inhibitory effect on actin polymerization.

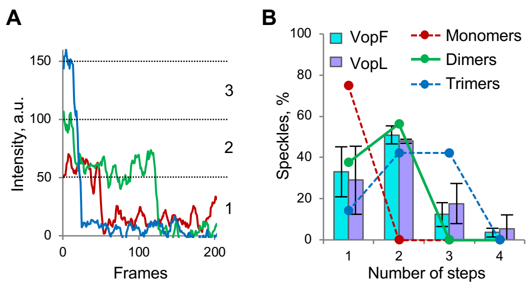

Fig. S4. VopF and VopL form homodimers inside the cell. Analysis of stepwise photobleaching [n=3 cells (100 speckles per cell; total 300) for each VopF and VopL] as described in Methods. Graph in **A** shows representative examples of 1-, 2-, and occasional 3-step bleaching events. Graph in **B** illustrates quantified bleaching events (shown as columns; mean ± SD) in comparison to binomial distribution models for monomeric, dimeric, and trimeric states (shown as lines).

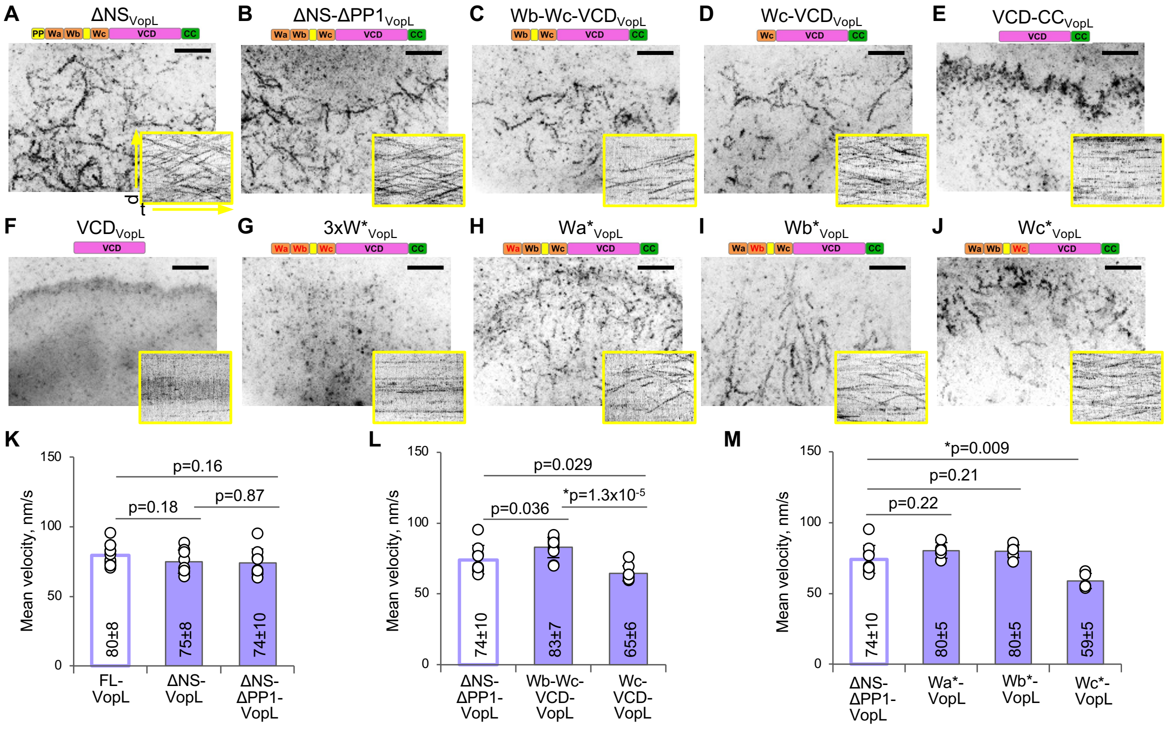

Fig. S5. WH2 domains enable processive motility of the dimeric VopL VCD. (**A-J**) Transiently transfected spreading XTC cells expressing very low (*i.e.*, suitable for single-molecule analysis) levels of EGFP-tagged constructs were selected for TIRF SiMS imaging. Maximum intensity projections of TIRF SiMS time-lapse images illustrate 1) trajectories of moving speckles (**A-D** and **H-J**), 2) mainly diffused localization (**F**), or 3) stationary speckles (**E** and **G**); scale bars are 5 µm. Kymographs are shown in yellow boxes; d – distance (15 µm), t – time (121 s). (**K-M**) Graphs show velocities of processively moving constructs calculated using TrackMate analysis. Numerical data presented as mean ± SD. Number of quantified cells and tracks: ΔNS-VopL, n=9 cells (229-980 tracks per cell; total 4410); ΔNS-ΔPP1-VopL, n=9 cells (309-631 tracks per cell; total 4444); Wb-Wc-VCD-VopL, n=11 cells (76-355 tracks per cell; total 2349); Wc-VCD-VopL, n=8 cells (158-612 tracks per cell; total 2553); Wa*-VopL, n=5 cells (333-589 tracks per cell; total 2056); Wb*-VopL, n=6 cells (160-589 tracks per cell; total 1903); Wc*-VopL, n=5 cells (113-460 tracks per cell; total 1076).

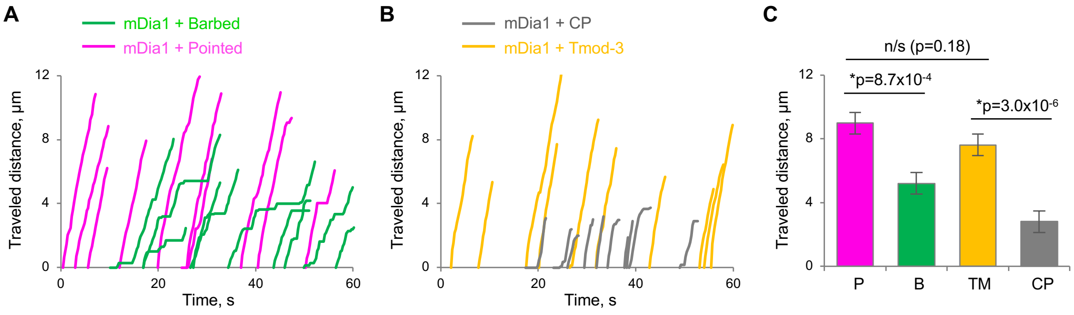

Fig. S6. Processive motility of constitutively active mDia1 formin is affected by blocking barbed filament ends. Co-expression of EGFP-mDia1-ΔN3 with one of the following mCherry-tagged constructs: (**A**) "barbed"-surface actin mutant (Barbed) or "pointed"-surface actin mutant (Pointed), (**B**) capping protein (CP) or tropomodulin-3 (Tmod-3). Cells expressing both, moderate to low levels of mCherry-tagged constructs (*i.e.*, either actin mutants, CP, or Tmod-3) and very low (*i.e.*, suitable for single-molecule analysis) levels of EGFP-VopF were selected for TIRF SiMS imaging. EGFP-mDia1-ΔN3 speckle tracks (n=10) were plotted as traveled distance as a function of time. See also movie S10. (**C**) Mean track lengths (± SD) of EGFP-mDia1-ΔN3 speckles (n=10) in the presence of "pointed"-surface actin mutant (P), "barbed"-surface actin mutant (B), tropomodulin-3 (TM), or capping protein (CP).

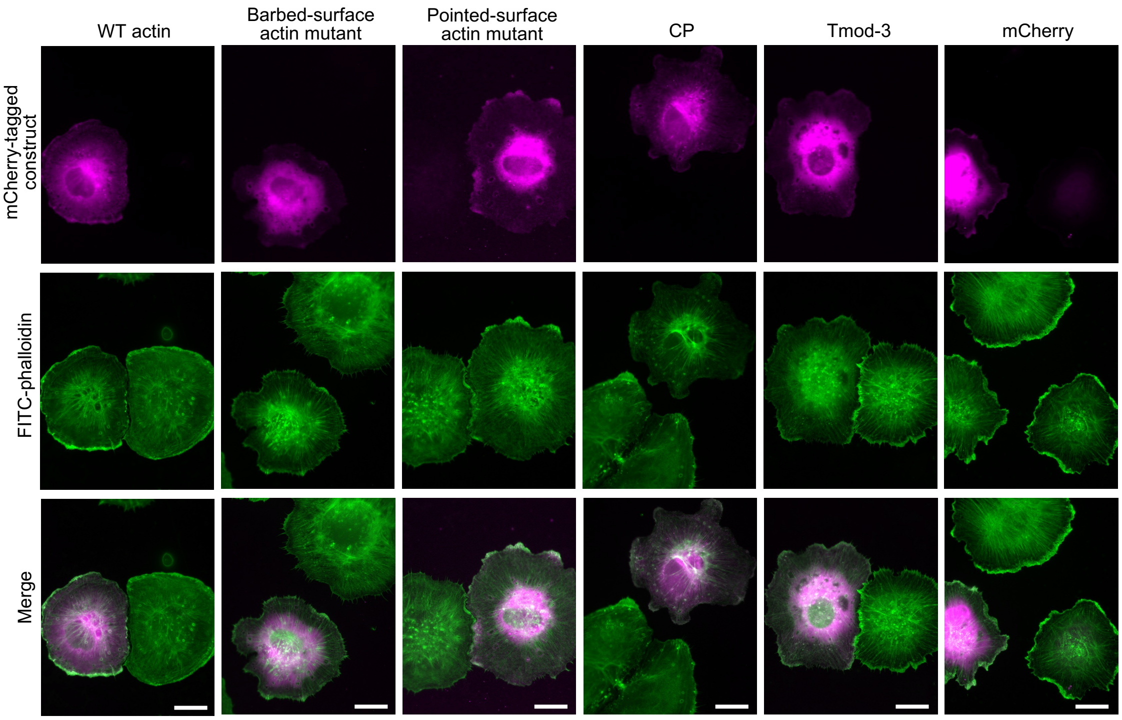

Fig. S7. Moderate expression levels of the actin mutants, CP, and Tmod-3 do not affect actin cytoskeleton and cell morphology. Representative images of spreading XTC cells transfected with one of the following mCherry-tagged constructs: WT actin, barbed-surface actin mutant, pointed-surface actin mutant, capping protein (CP), tropomodulin-3 (Tmod-3), or mCherry only and contra stained with FITC-phalloidin. Scale bars are 20 µm.

Movie captions S1 to S15

Movie S1. VopF and VopL induce protrusions in U2OS cells.
U2OS cells co-transfected with mCherry-Utr-CH (utrophin calponin-homology domain used as an F-actin marker) and either EGFP alone, EGFP-VopF, or EGFP-VopL. Double transfected cells expressing moderate levels of mCherry- and EGFP-tagged constructs were selected for imaging. Scale bars are 10 µm.

Movie S2. VopF- and VopL-induced cellular protrusions resemble those induced by constitutively active mDia2 formin.
XTC cells expressing low levels of either EGFP-VopF, EGFP-VopL, or EGFP-mDia2-ΔN3. Scale bars are 5 µm.

Movie S3. VopF and VopL move persistently inside the cell.
XTC cells expressing very low (*i.e.*, suitable for single-molecule analysis) levels of EGFP-VopF or EGFP-VopL were selected for TIRF SiMS imaging. Scale bars are 5 µm.

Movie S4. Single particle tracking of VopF using TrackMate analysis.
Automated TrackMate analysis of VopF speckles as described in the fig. S2A-D legend and Methods.

Movie S5. Microtubule- and myosin-targeting drugs do not affect processive motility of VopF speckles.
TIRF SiMS imaging of XTC cells expressing very low levels of EGFP-VopF before (0 min) and after (30 and 60 min) the addition of the indicated drugs: 10 µM nocodazole, 25 µM blebbistatin (Bleb), 25 µM MyoVin1. For each treatment, the same cell is imaged before and after the drug treatment. Scale bars are 5 µm.

Movie S6. Actin-targeting drugs at 1-µM concentrations halt processive motility of VopF speckles.
TIRF SiMS imaging of XTC cells expressing very low (*i.e.*, suitable for single-molecule analysis) levels of EGFP-VopF in the absence or presence of 1 µM of the indicated actin-targeting drugs: Jasp, jasplakinolide; Swin, swinholide A; CytD, cytochalasin D; LatA, latrunculin A. For each treatment, time-lapse images of the same cells are shown before (upper row) and 5 min after adding the drugs (lower row). Scale bars are 5 µm.

Movie S7. At 1-nM concentration, only latrunculin A inhibits processive motility of VopF speckles.
TIRF SiMS imaging of XTC cells expressing very low levels of EGFP-VopF in the absence or presence of 1 nM of the indicated actin-targeting drugs: Jasp, jasplakinolide; Swin, swinholide A; CytD, cytochalasin D; LatA, latrunculin A. Time-lapse images were taken 30 min after the addition of Jasp, Swin, and CytD, and 5 min after the addition of LatA. Scale bars are 5 µm.

Movie S8. LatA at 1-nM concentration potently inhibits processive motility of VopL speckles.

TIRF SiMS imaging of an XTC cell expressing very low levels of EGFP-VopL before (-100 s to 0 s) and after adding 1 nM latrunculin A (1 s to 240 s, LatA, 1nM). Scale bars are 5 μm. See also Fig. 4A,B.

Movie S9. Blocking pointed filament ends inhibits processive motility of VopL speckles.

TIRF SiMS imaging of XTC cells expressing very low levels of EGFP-VopL. The cells are co-expressing one of the following mCherry-tagged constructs at moderate to low levels: "barbed"-surface actin mutant (Barbed*), "pointed"-surface actin mutant (Pointed*), capping protein (CP), or tropomodulin-3 (Tmod-3). Scale bars are 5 μm.

Movie S10. Processive motility of constitutively active mDia1 formin is affected by blocking barbed filament ends.

TIRF SiMS imaging of XTC cells expressing very low levels of EGFP-mDia1-ΔN3. The cells are co-expressing one of the following mCherry-tagged constructs at moderate to low levels: "barbed"-surface actin mutant (Barbed*), "pointed"-surface actin mutant (Pointed*), capping protein (CP), or tropomodulin-3 (Tmod-3). Scale bars are 5 μm. See also fig. S6.

Movie S11. Processive movement of VopF can be reconstituted *in vitro*.

In vitro reconstituted TIRFM imaging of free unanchored SNAP-549-ΔNS-VopF (4 nM) in the presence of 10 μM of unlabeled actin, 12 μM of unlabeled PFN1, and 20 nM of unlabeled CP. The right panel shows the same tame-lapse images (as in the left panel) with the processively moving SNAP-549-ΔNS-VopF speckles tracked using TrackMate. Scale bars are 5 μm.

Movie S12. VopF is a processive actin polymerase.

Small fluorescent filaments were nucleated by flowing 1.5 μM Alexa-488-labelled actin monomers into the mf-TIRFM chamber with 6xHis-SNAP-ΔNS-VopF-functionalized coverslip surface, followed by flowing in a mixture of 10 μM unlabeled actin with 15 μM PFN. See also Fig. 5C,D.

Movie S13. Processive pointed-end actin elongation by VopF.

Small fluorescent filaments were nucleated by flowing 1.5 μM Alexa-488-labelled actin monomers into the mf-TIRFM chamber with SNAP-biotin-ΔNS-VopF-functionalized coverslip surface, followed by flowing in a mixture of 10 μM unlabeled actin with 15 μM PFN and 5 nM SNAP-549-CP. White arrowheads indicate the points of anchoring of the nucleated filaments (via the surface-attached SNAP-biotin-ΔNS-VopF). Cian arrowheads indicate the barbed ends (capped by SNAP-549-CP) of actin filaments continuing to grow at the points of anchoring. See also Fig. 5E-G.

Movie S14. Unassisted elongation of free pointed ends. To determine the rate of unassisted elongation of F-actin free pointed ends (Fig. 5D), labeled pre-formed filaments were captured on the coverslip surface by SNAP-biotin-CP. The filaments were then exposed to alternating flows of

10 μM Alexa488-G-actin with 15 μM PFN (for 55 s out of every minute) and 10 μM unlabeled G-actin with 15 μM PFN (for 5 s every min). TIRFM images were acquired once every minute during the period when unlabeled actin was flowing. To prevent the surface attachment of spontaneously nucleated filaments from the flow, 1 μM of free CP was added to both, labeled and unlabeled actin/PFN mixtures.

Movie S15. Actin retrograde flow is abrogated by VopF/L. TIRF SiMS imaging of XTC cells expressing very low levels of TagRFPT-actin. The cells are co-expressing moderate to low levels of either EGFP, EGFP-VopF, or EGFP-VopL. Scale bars are 5 μm.

REFERENCES AND NOTES

1. T. Svitkina, The actin cytoskeleton and actin-based motility. *Cold Spring Harb. Perspect. Biol.* **10**, a018267 (2018).

2. A. Narita, T. Oda, Y. Maéda, Structural basis for the slow dynamics of the actin filament pointed end. *EMBO J.* **30**, 1230–1237 (2011).

3. T. D. Pollard, Actin and actin-binding proteins. *Cold Spring Harb. Perspect. Biol.* **8**, a018226 (2016).

4. M. F. Carlier, S. Shekhar, Global treadmilling coordinates actin turnover and controls the size of actin networks. *Nat. Rev. Mol. Cell Biol.* **18**, 389–401 (2017).

5. T. Kotila, H. Wioland, G. Enkavi, K. Kogan, I. Vattulainen, A. Jégou, G. Romet-Lemonne, P. Lappalainen, Mechanism of synergistic actin filament pointed end depolymerization by cyclase-associated protein and cofilin. *Nat. Commun.* **10**, 5320 (2019).

6. S. Shekhar, J. Chung, J. Kondev, J. Gelles, B. L. Goode, Synergy between cyclase-associated protein and cofilin accelerates actin filament depolymerization by two orders of magnitude. *Nat. Commun.* **10**, 5319 (2019).

7. V. W. Tang, A. V. Nadkarni, W. M. Brieher, Catastrophic actin filament bursting by cofilin, Aip1, and coronin. *J. Biol. Chem.* **295**, 13299–13313 (2020).

8. M.-F. Carlier, J. Pernier, P. Montaville, S. Shekhar, S. Kühn; Cytoskeleton Dynamics and Motility group, Control of polarized assembly of actin filaments in cell motility. *Cell. Mol. Life Sci.* **72**, 3051–3067 (2015).

9. A. L. Hatch, P. S. Gurel, H. N. Higgs, Novel roles for actin in mitochondrial fission. *J. Cell Sci.* **127**, 4549–4560 (2014).

10. M. Izdebska, W. Zielińska, M. Halas-Wiśniewska, A. Grzanka, Involvement of actin in autophagy and autophagy-dependent multidrug resistance in cancer. *Cancers (Basel)* **11**, 1209 (2019).

11. V. Papalazarou, L. M. Machesky, The cell pushes back: The Arp2/3 complex is a key orchestrator of cellular responses to environmental forces. *Curr. Opin. Cell Biol.* **68**, 37–44 (2021).

12. R. Gorelik, C. Yang, V. Kameswaran, R. Dominguez, T. Svitkina, Mechanisms of plasma membrane targeting of formin mDia2 through its amino terminal domains. *Mol. Biol. Cell* **22**, 189–201 (2011).

13. A. Seth, C. Otomo, M. K. Rosen, Autoinhibition regulates cellular localization and actin assembly activity of the diaphanous-related formins FRLalpha and mDia1. *J. Cell Biol.* **174**, 701–713 (2006).

14. J. Tittel, T. Welz, A. Czogalla, S. Dietrich, A. Samol-Wolf, M. Schulte, P. Schwille, T. Weidemann, E. Kerkhoff, Membrane targeting of the Spir.formin actin nucleator complex requires a sequential handshake of polar interactions. *J. Biol. Chem.* **290**, 6428–6444 (2015).

15. M. Izadi, E. Seemann, D. Schlobinski, L. Schwintzer, B. Qualmann, M. M. Kessels, Functional interdependence of the actin nucleator Cobl and Cobl-like in dendritic arbor development. *eLife* **10**, e67718 (2021).

16. M. A. Titus, Myosin-driven intracellular transport. *Cold Spring Harb. Perspect. Biol.* **10**, a021972 (2018).

17. R. Nambiar, R. E. McConnell, M. J. Tyska, Control of cell membrane tension by myosin-I. *Proc. Natl. Acad. Sci. U.S.A.* **106**, 11972–11977 (2009).

18. D. J. Kast, A. L. Zajac, E. L. Holzbaur, E. M. Ostap, R. Dominguez, WHAMM directs the Arp2/3 complex to the ER for autophagosome biogenesis through an actin comet tail mechanism. *Curr. Biol.* **25**, 1791–1797 (2015).

19. F. Korobova, V. Ramabhadran, H. N. Higgs, An actin-dependent step in mitochondrial fission mediated by the ER-associated formin INF2. *Science* **339**, 464–467 (2013).

20. C. J. Merrifield, S. E. Moss, C. Ballestrem, B. A. Imhof, G. Giese, I. Wunderlich, W. Almers, Endocytic vesicles move at the tips of actin tails in cultured mast cells. *Nat. Cell Biol.* **1**, 72–74 (1999).

21. M. T. Butler, J. B. Wallingford, Planar cell polarity in development and disease. *Nat. Rev. Mol. Cell Biol.* **18**, 375–388 (2017).

22. E. Rodriguez-Boulan, I. G. Macara, Organization and execution of the epithelial polarity programme. *Nat. Rev. Mol. Cell Biol.* **15**, 225–242 (2014).

23. M. Inaki, T. Sasamura, K. Matsuno, Cell chirality drives left-right asymmetric morphogenesis. *Front. Cell Dev. Biol.* **6**, 34 (2018).

24. M. K. Hoelzle, T. Svitkina, The cytoskeletal mechanisms of cell-cell junction formation in endothelial cells. *Mol. Biol. Cell* **23**, 310–323 (2012).

25. C. S. Tran, Y. Eran, T. R. Ruch, D. M. Bryant, A. Datta, P. Brakeman, A. Kierbel, T. Wittmann, R. J. Metzger, K. E. Mostov, J. N. Engel, Host cell polarity proteins participate in innate immunity to *Pseudomonas aeruginosa* infection. *Cell Host Microbe* **15**, 636–643 (2014).

26. P. M. Colonne, C. G. Winchell, D. E. Voth, Hijacking host cell highways: Manipulation of the host actin cytoskeleton by obligate intracellular bacterial pathogens. *Front. Cell. Infect. Microbiol.* **6**, 107 (2016).

27. F. Navarro-Garcia, A. Serapio-Palacios, P. Ugalde-Silva, G. Tapia-Pastrana, L. Chavez-Dueñas, Actin cytoskeleton manipulation by effector proteins secreted by diarrheagenic *Escherichia coli* pathotypes. *Biomed. Res. Int.* **2013**, 374395 (2013).

28. M. de Souza Santos, D. Salomon, K. Orth, T3SS effector VopL inhibits the host ROS response, promoting the intracellular survival of *Vibrio parahaemolyticus*. *PLOS Pathog.* **13**, e1006438 (2017).

29. A. D. B. Liverman, H.-C. Cheng, J. E. Trosky, D. W. Leung, M. L. Yarbrough, D. L. Burdette, M. K. Rosen, K. Orth, Arp2/3-independent assembly of actin by *Vibrio* type III effector VopL. *Proc. Natl. Acad. Sci. U.S.A.* **104**, 17117–17122 (2007).

30. V. C. Tam, D. Serruto, M. Dziejman, W. Brieher, J. J. Mekalanos, A type III secretion system in *Vibrio cholerae* translocates a formin/spire hybrid-like actin nucleator to promote intestinal colonization. *Cell Host Microbe* **1**, 95–107 (2007).

31. V. C. Tam, M. Suzuki, M. Coughlin, D. Saslowsky, K. Biswas, W. I. Lencer, S. M. Faruque, J. J. Mekalanos, Functional analysis of VopF activity required for colonization in *Vibrio cholerae*. *MBio* **1**, e00289-10 (2010).

32. T. A. Burke, A. J. Harker, R. Dominguez, D. R. Kovar, The bacterial virulence factors VopL and VopF nucleate actin from the pointed end. *J. Cell Biol.* **216**, 1267–1276 (2017).

33. S. Namgoong, M. Boczkowska, M. J. Glista, J. D. Winkelman, G. Rebowski, D. R. Kovar, R. Dominguez, Mechanism of actin filament nucleation by *Vibrio* VopL and implications for tandem W domain nucleation. *Nat. Struct. Mol. Biol.* **18**, 1060–1067 (2011).

34. R. Dominguez, The WH2 domain and actin nucleation: Necessary but insufficient. *Trends Biochem. Sci.* **41**, 478–490 (2016).

35. J. Pernier, J. Orban, B. S. Avvaru, A. Jégou, G. Romet-Lemonne, B. Guichard, M. F. Carlier, Dimeric WH2 domains in *Vibrio* VopF promote actin filament barbed-end uncapping and assisted elongation. *Nat. Struct. Mol. Biol.* **20**, 1069–1076 (2013).

36. B. Yu, H. C. Cheng, C. A. Brautigam, D. R. Tomchick, M. K. Rosen, Mechanism of actin filament nucleation by the bacterial effector VopL. *Nat. Struct. Mol. Biol.* **18**, 1068–1074 (2011).

37. J. A. Zahm, S. B. Padrick, Z. Chen, C. W. Pak, A. A. Yunus, L. Henry, D. R. Tomchick, Z. Chen, M. K. Rosen, The bacterial effector VopL organizes actin into filament-like structures. *Cell* **155**, 423–434 (2013).

38. C. Higashida, T. Miyoshi, A. Fujita, F. Oceguera-Yanez, J. Monypenny, Y. Andou, S. Narumiya, N. Watanabe, Actin polymerization-driven molecular movement of mDia1 in living cells. *Science* **303**, 2007–2010 (2004).

39. D. R. Kovar, E. S. Harris, R. Mahaffy, H. N. Higgs, T. D. Pollard, Control of the assembly of ATP- and ADP-actin by formins and profilin. *Cell* **124**, 423–435 (2006).

40. N. Watanabe, T. J. Mitchison, Single-molecule speckle analysis of actin filament turnover in lamellipodia. *Science* **295**, 1083–1086 (2002).

41. N. Watanabe, Fluorescence single-molecule imaging of actin turnover and regulatory mechanisms. *Methods Enzymol.* **505**, 219–232 (2012).

42. J. Schindelin, I. Arganda-Carreras, E. Frise, V. Kaynig, M. Longair, T. Pietzsch, S. Preibisch, C. Rueden, S. Saalfeld, B. Schmid, J.Y. Tinevez, D. J. White, V. Hartenstein, K. Eliceiri, P. Tomancak, A. Cardona, Fiji: An open-source platform for biological-image analysis. *Nat. Methods* **9**, 676–682 (2012).

43. D. Ershov, M.-S. Phan, J. W. Pylvänäinen, S. U. Rigaud, L. L. Blanc, A. C.-Orszag, J. R. W. Conway, R. F. Laine, N. H. Roy, D. Bonazzi, G. Duménil, G. Jacquemet, J.-Y. Tinevez, Bringing TrackMate into the era of machine-learning and deep-learning. bioRxiv 2021.09.03.458852 [**Preprint**]. 20 September 2021. https://doi.org/10.1101/2021.09.03.458852.

44. J. Y. Tinevez, N. Perry, J. Schindelin, G. M. Hoopes, G.D. Reynolds, E. Laplantine, S. Y. Bednarek, S.L. Shorte, K. W. Eliceiri, TrackMate: An open and extensible platform for single-particle tracking. *Methods* **115**, 80–90 (2017).

45. E. Kudryashova, D. B. Heisler, B. Williams, A. J. Harker, K. Shafer, M. E. Quinlan, D. R. Kovar, D. Vavylonis, D. S. Kudryashov, Actin cross-linking toxin is a universal inhibitor of tandem-organized and oligomeric g-actin binding proteins. *Curr. Biol.* **28**, 1536–1547.e9 (2018).

46. V. C. Coffman, J. Q. Wu, Counting protein molecules using quantitative fluorescence microscopy. *Trends Biochem. Sci.* **37**, 499–506 (2012).

47. A. J. Harker, H. H. Katkar, T. C. Bidone, F. Aydin, G. A. Voth, D. A. Applewhite, D. R. Kovar, Ena/VASP processive elongation is modulated by avidity on actin filaments bundled by the filopodia cross-linker fascin. *Mol. Biol. Cell* **30**, 851–862 (2019).

48. M. R. Bubb, A. M. Senderowicz, E. A. Sausville, K. L. Duncan, E. D. Korn, Jasplakinolide, a cytotoxic natural product, induces actin polymerization and competitively inhibits the binding of phalloidin to F-actin. *J. Biol. Chem.* **269**, 14869–14871 (1994).

49. M. R. Bubb, I. Spector, B. B. Beyer, K. M. Fosen, Effects of jasplakinolide on the kinetics of actin polymerization. An explanation for certain in vivo observations. *J. Biol. Chem.* **275**, 5163–5170 (2000).

50. M. R. Bubb, I. Spector, A. D. Bershadsky, E. D. Korn, Swinholide A is a microfilament disrupting marine toxin that stabilizes actin dimers and severs actin filaments. *J. Biol. Chem.* **270**, 3463–3466 (1995).

51. J. A. Cooper, Effects of cytochalasin and phalloidin on actin. *J. Cell Biol.* **105**, 1473–1478 (1987).

52. U. B. Nair, P. B. Joel, Q. Wan, S. Lowey, M. A. Rould, K. M. Trybus, Crystal structures of monomeric actin bound to cytochalasin D. *J. Mol. Biol.* **384**, 848–864 (2008).

53. M. Coue, S. L. Brenner, I. Spector, E. D. Korn, Inhibition of actin polymerization by latrunculin A. *FEBS Lett.* **213**, 316–318 (1987).

54. E. G. Yarmola, T. Somasundaram, T. A. Boring, I. Spector, M. R. Bubb, Actin-latrunculin A structure and function. Differential modulation of actin-binding protein function by latrunculin A. *J. Biol. Chem.* **275**, 28120–28127 (2000).

55. W. M. Morton, K. R. Ayscough, P. J. McLaughlin, Latrunculin alters the actin-monomer subunit interface to prevent polymerization. *Nat. Cell Biol.* **2**, 376–378 (2000).

56. P. B. Joel, P. M. Fagnant, K. M. Trybus, Expression of a nonpolymerizable actin mutant in Sf9 cells. *Biochemistry* **43**, 11554–11559 (2004).

57. J. Funk, F. Merino, M. Schaks, K. Rottner, S. Raunser, P. Bieling, A barbed end interference mechanism reveals how capping protein promotes nucleation in branched actin networks. *Nat. Commun.* **12**, 5329 (2021).

58. A. Yamashita, K. Maeda, Y. Maeda, Crystal structure of CapZ: Structural basis for actin filament barbed end capping. *EMBO J.* **22**, 1529–1538 (2003).

59. J. N. Rao, Y. Madasu, R. Dominguez, Mechanism of actin filament pointed-end capping by tropomodulin. *Science* **345**, 463–467 (2014).

60. A. Jégou, T. Niedermayer, J. Orbán, D. Didry, R. Lipowsky, M.-F. Carlier, G. Romet-Lemonne, Individual actin filaments in a microfluidic flow reveal the mechanism of ATP hydrolysis and give insight into the properties of profilin. *PLOS Biol.* **9**, e1001161 (2011).

61. S. Shekhar, Microfluidics-assisted TIRF imaging to study single actin filament dynamics. *Curr. Protoc. Cell Biol.* **77**, 12.13.11–12.13.24 (2017).

62. M. H. Symons, T. J. Mitchison, Control of actin polymerization in live and permeabilized fibroblasts. *J. Cell Biol.* **114**, 503–513 (1991).

63. N. Ofer, E. Abu Shah, K. Keren, Differential mapping of the free barbed and pointed ends of actin filaments in cells. *Cytoskeleton (Hoboken)* **71**, 341–350 (2014).

64. S. Shekhar, G. J. Hoeprich, J. Gelles, B. L. Goode, Twinfilin bypasses assembly conditions and actin filament aging to drive barbed end depolymerization. *J. Cell Biol.* **220**, e202006022 (2021).

65. J. R. Christensen, G. M. Hocky, K. E. Homa, A. N. Morganthaler, S. E. Hitchcock-DeGregori, G. A. Voth, D. R. Kovar, Competition between tropomyosin, fimbrin, and ADF/Cofilin drives their sorting to distinct actin filament networks. *eLife* **6**, e23152 (2017).

66. B. Johnson, P. McConnell, A. G. Kozlov, M. Mekel, T. M. Lohman, M. L. Gross, G. K. Amarasinghe, J. A. Cooper, Allosteric coupling of CARMIL and V-1 binding to capping protein revealed by hydrogen-deuterium exchange. *Cell Rep.* **23**, 2795–2804 (2018).

67. C. Y. Lim, X. Bi, D. Wu, J. B. Kim, P. W. Gunning, W. Hong, W. Han, Tropomodulin3 is a novel Akt2 effector regulating insulin-stimulated GLUT4 exocytosis through cortical actin remodeling. *Nat. Commun.* **6**, 5951 (2015).

68. E. B. Merriam, M. Millette, D. C. Lumbard, W. Saengsawang, T. Fothergill, X. Hu, L. Ferhat, E. W. Dent, Synaptic regulation of microtubule dynamics in dendritic spines by calcium, F-actin, and drebrin. *J. Neurosci.* **33**, 16471–16482 (2013).

69. B. M. Burkel, G. von Dassow, W. M. Bement, Versatile fluorescent probes for actin filaments based on the actin-binding domain of utrophin. *Cell Motil. Cytoskeleton* **64**, 822–832 (2007).

70. A. Bisaria, A. Hayer, D. Garbett, D. Cohen, T. Meyer, Membrane-proximal F-actin restricts local membrane protrusions and directs cell migration. *Science* **368**, 1205–1210 (2020).

71. J. D. Pardee, J. A. Spudich, Purification of muscle actin. *Methods Enzymol.* **85** Pt B, 164–181 (1982).

72. J. A. Cooper, T. D. Pollard, Methods to measure actin polymerization. *Methods Enzymol.* **85** Pt B, 182–210 (1982).

73. L. K. Doolittle, M. K. Rosen, S. B. Padrick, Measurement and analysis of in vitro actin polymerization. *Methods Mol. Biol.* **1046**, 273–293 (2013).

74. J. Lu, T. D. Pollard, Profilin binding to poly-L-proline and actin monomers along with ability to catalyze actin nucleotide exchange is required for viability of fission yeast. *Mol. Biol. Cell* **12**, 1161–1175 (2001).

75. C. J. Knoot, V. M. Purpero, J. D. Lipscomb, Crystal structures of alkylperoxo and anhydride intermediates in an intradiol ring-cleaving dioxygenase. *Proc. Natl. Acad. Sci. U.S.A.* **112**, 388–393 (2015).

76. D. Breitsprecher, R. Jaiswal, J. P. Bombardier, C. J. Gould, J. Gelles, B. L. Goode, Rocket launcher mechanism of collaborative actin assembly defined by single-molecule imaging. *Science* **336**, 1164–1168 (2012).

77. M. H. Ulbrich, E. Y. Isacoff, Subunit counting in membrane-bound proteins. *Nat. Methods* **4**, 319–321 (2007).

78. C. E. Aitken, R. A. Marshall, J. D. Puglisi, An oxygen scavenging system for improvement of dye stability in single-molecule fluorescence experiments. *Biophys. J.* **94**, 1826–1835 (2008).

79. P. Hotulainen, P. Lappalainen, Stress fibers are generated by two distinct actin assembly mechanisms in motile cells. *J. Cell Biol.* **173**, 383–394 (2006).

80. D. W. Goddette, C. Frieden, Actin polymerization. The mechanism of action of cytochalasin D. *J. Biol. Chem.* **261**, 15974–15980 (1986).

81. V. A. Klenchin, R. King, J. Tanaka, G. Marriott, I. Rayment, Structural basis of swinholide A binding to actin. *Chem. Biol.* **12**, 287–291 (2005).
